# Changes in Cognition, Depressive Symptoms and Functional Limitation Before and After Incident Chronic Kidney Disease: A Three-Cohort Longitudinal Study

**DOI:** 10.3390/healthcare14193215

**Published:** 2026-09-29

**Authors:** Zhengzhong Shen, Qun Xiao, Weicheng Huang, Gang Peng

**Affiliations:** 1 Department of Oral and Maxillofacial Surgery, Center of Stomatology, Xiangya Hospital of Central South University, Changsha 410011, China; 2 Department of Neurosurgery, Xiangya Hospital of Central South University, Changsha 410011, China; drxiaoqun@csu.edu.cn (Q.X.); huangweicheng@csu.edu.cn (W.H.)

**Keywords:** chronic kidney disease, cognitive decline, depressive symptoms, functional limitation, longitudinal trajectories, multiple imputation, ageing cohorts, CHARLS/HRS/SHARE

## Abstract

**Background:** Chronic kidney disease (CKD) is linked to cognitive, psychological and functional decline, yet few studies track these domains together around the first detection of incident disease. We examined three longitudinal outcomes while preserving observed CKD onset and ordinary study time for participants who remained free of CKD. **Methods:** We analysed harmonised longitudinal survey and biomarker data from CHARLS (China, 2011–2018), HRS (United States, 2006–2016) and SHARE (Europe and Israel, 2015–2022) among adults aged 50 years or older. The main comparison included 75,044 participants (2480 with incident CKD and 72,564 free of CKD). Global cognition, depressive symptoms and functional limitation were three prespecified co-primary outcome domains, not a composite. Missing baseline covariates were imputed separately by cohort with multiple imputation by chained equations (MICE; m = 20, 20 iterations). Random-intercept restricted maximum likelihood (REML) mixed models (Model 1 and Model 2) were fitted in each completed data set and combined with Rubin’s rules. Model 2 adjusted for seven baseline chronic disease indicators, body mass index, smoking and alcohol use. For each outcome, inference focused on the change in the annual outcome trajectory associated with incident CKD occurrence (the trajectory change estimate), with Benjamini–Hochberg correction across the three Model 2 tests. **Results:** Rubin-pooled Model 2 trajectory change estimates were global cognition, −0.019 (−0.031 to −0.007) (BH q = 0.003); depressive symptoms, 0.018 (0.005 to 0.032) (BH q = 0.008); functional limitation, 0.075 (0.063 to 0.087) (BH q < 0.001), in standardised outcome units per year. An exploratory joint study-time module identified three trajectory classes and compared class membership by CKD status. Lagged Cox models used CKD status at the interval start for four non-frailty, visit-detected proxy endpoints. **Conclusions:** Incident CKD was associated with directionally and quantitatively distinct longitudinal changes in cognition, depressive symptoms and functional limitation. These results are adjusted associations, not instantaneous causal effects. Joint trajectory classes and lagged event models were exploratory; frailty index and stacked three-domain GEE analyses were supplementary.

## 1. Introduction

Chronic kidney disease (CKD) is a major source of morbidity worldwide and becomes more common with age [1,2,3]. Its consequences extend beyond reduced filtration: older adults with CKD often carry vascular and metabolic comorbidities and may experience changes in cognition, mood, mobility and independence. These outcomes matter clinically because they shape treatment capacity, quality of life and the ability to live independently.

Separate lines of literature link CKD to cognitive impairment, depressive symptoms and impaired physical function [1,2,3,4,5,6]. Deficit accumulation frailty provides an additional summary of vulnerability [7], but frailty indices often overlap with cognition, mood, disability and disease covariates. Studying each domain alone can miss whether several systems worsen over a similar period; combining overlapping constructs without qualification can overstate the coherence of a multisystem signal.

Longitudinal multisystem evidence remains limited. Many studies evaluate prevalent or baseline CKD, analyse outcomes separately or do not distinguish the level observed at the first CKD-positive visit from subsequent change. Because both exposure and outcome are assessed at spaced visits, observed onset is an interval-censored detection time rather than the exact biological onset. Event-centred designs in other chronic diseases illustrate the value of separating pre-event slopes, occurrence-level contrasts and post-event trajectory change [8]. To our knowledge, an analogous three-cohort analysis organised around incident CKD, without assigning pseudo-onset dates to CKD-free participants, has not been reported.

We therefore examined whether incident CKD was associated with changes in global cognition, depressive symptoms and functional limitation. Primary models preserved ordinary study time for CKD-free participants, separated the model-level change at incident CKD occurrence from the change in the annual outcome trajectory associated with incident CKD occurrence, and fitted every cohort-specific MICE completion before Rubin pooling. Three-domain study-time trajectory classes and lagged visit-detected event models were exploratory extensions. Because the deficit accumulation index overlaps the primary domains and adjustment covariates, FI14 and a reduced-overlap FI10 variant were analysed only as supplementary outcomes.

## 2. Methods

### 2.1. Study Design and Cohorts

We used harmonised longitudinal data from CHARLS, HRS and SHARE and retained locked creatinine-, cystatin-C- and self-report CKD definitions [9,10,11,12,13]. Observation windows were 2011–2018 in CHARLS, 2006–2016 in HRS and 2015–2022 in SHARE. Adults aged 50 years or older entered the incident CKD versus CKD-free comparison; prevalent CKD was excluded. Participants free of CKD were not assigned pseudo-onset dates. Analytic flow and cohort-specific kidney source definitions are documented in Tables S1 and S2.

### 2.2. Outcomes and Covariates

Global cognition, depressive symptoms and functional limitation were three prespecified co-primary outcome domains and were not combined into a composite outcome. The three domains represent distinct cognitive, psychological and functional constructs, each central to the study question. No composite score or weighting scheme was prespecified or validated for this analysis. Cognition was scored higher—better; the other outcomes were higher—worse.

Seven baseline disease indicators entered Model 2 separately: hypertension, diabetes, cancer, lung disease, heart disease, stroke and arthritis. Their count was descriptive only and was neither imputed nor used as an adjustment covariate. These baseline indicators were retained to control pre-existing comorbidity without conditioning on disease changes occurring after CKD ascertainment that could be mediators. The formal analysis used baseline disease indicators replicated at every retained visit. The requested time-updated sensitivity analysis was not undertaken; constancy in these completed files does not establish that longitudinal disease histories are unavailable in the original cohorts (Table S4).

The 14-item frailty index (FI14) and reduced-overlap 10-item frailty index (FI10) were supplementary because FI14 includes depression, activities of daily living (ADL) and instrumental activities of daily living (IADL) items, cognition or dementia, disease, body mass index and smoking components.

### 2.3. Multiple Imputation

Within each cohort, missing baseline covariates were imputed by multivariate imputation by chained equations (MICE) with 20 imputations and 20 iterations [14,15]. Global cognition, depressive symptoms, functional limitation, CKD exposure, timing variables, identifiers and events were protected from imputation. Every scientific model was fitted separately in all completed data sets; estimates and variances, not completed data sets, were combined with Rubin’s rules [16], with small-sample degrees of freedom following Barnard and Rubin where applicable [17]. Cohort-specific missingness and imputation diagnostics are reported in Table S1. For supplementary analyses, FI14 and FI10 were recalculated within each completion from completed components.

### 2.4. Three-Outcome Longitudinal Models

For each co-primary outcome, we fitted a random-intercept REML model without pseudo-onset assignment. Model 1 included study time, cohort and cohort-by-time, a person-level incident CKD indicator and its interaction with study time, a model-level change term at incident CKD occurrence, the change in the annual outcome trajectory associated with incident CKD occurrence, and age, sex and education. Model 2 additionally included the seven baseline disease indicators separately, body mass index, smoking and alcohol use. For each Model 2 estimate, the reported *p* value was the unadjusted Wald test for the trajectory change coefficient; the three co-primary tests formed the Benjamini–Hochberg family and are reported as BH q values. Benjamini–Hochberg adjustment controls the false discovery rate within this three-test family. P values for descriptive comparisons of baseline characteristics are unadjusted. Holm-adjusted *p* values are used only within prespecified exploratory or supplementary families. The level-change term is a level contrast at the observed occurrence assessment; the trajectory change term is the change in annual trajectory among participants who developed CKD. Because assessments occurred at discrete visits, neither term identifies an instantaneous biological effect of kidney disease, and no CKD-free participant is assigned an artificial event date.

Prespecified sensitivity analyses examined sample and visit definitions, the contribution of the occurrence visit, follow-up window, time functional form, cohort interaction, leave-one-cohort-out stability, covariance structure, selection and visit observation weighting and CKD source restrictions. The reviewer-requested source-restricted primary structure models retained the Model 2 random-intercept REML trajectory change estimand for biomarker-defined CHARLS plus HRS and for HRS cystatin-C alone. Design and comparability are summarised in Table S3; primary estimand, source-restricted, cohort-specific and clinical translation results are reported in Table S4; marginal weighted analyses and weight diagnostics remain in Table S5.

The two-panel Model 2 trajectory display was constructed as follows. Panel A shows incident CKD trajectories on either side of CKD occurrence together with a CKD-free ordinary study time reference, each outcome on its native standardised score scale. Predictions were standardised to the incident-participant baseline covariate and cohort distribution. The median observed study time at incident CKD occurrence provides a shared display origin only and does not assign an event time to CKD-free participants. Panel B displays the primary trajectory change estimate as burden-oriented cumulative additional change relative to the projected continuation of the incident group’s pre-CKD trajectory. Every completion contributed estimates and variances before Rubin pooling.

### 2.5. Joint Trajectories and Event Models

The exploratory three-domain study-time profile uses the first three complete cognition, depressive symptom and functional limitation visits, vectorised k-means class construction and multinomial class membership models fitted in each imputed data set, with estimates pooled using Rubin’s rules. The multinomial class membership contrast is reported as a relative risk ratio (RRR). Class membership is compared between incident CKD and CKD-free participants but is not aligned to incident CKD occurrence; candidate solutions, class sizes and initialisation stability are reported in Table S6. Main exploratory start-stop Cox models were fitted for four non-frailty visit-detected proxy endpoints, with CKD status lagged to the interval start. Same-wave events were not classified as exposed. Endpoint definitions, complete estimates and proportional hazards diagnostics are reported in Table S7. The frailty proxy appears only in the supplement. These exploratory models use endpoint-specific risk sets and lagged exposure, so null cognitive event estimates do not negate the continuous cognition result. Likewise, the supplementary stacked GEE answers a marginal multi-outcome question and does not replace the primary conditional mixed models.

### 2.6. Supplementary Direct Joint Model

FI14 and reduced-overlap FI10 results are reported in Table S8. As a supplementary sensitivity analysis, we also fitted a direct three-domain stacked generalised estimating equation (GEE) model for correlated longitudinal outcomes [18,19], implemented in geepack [20]. Across imputations, the joint null that all three domain-specific trajectory change coefficients equal zero was evaluated with a multiparameter D1 test [21]. Cohort-specific GEE estimates were synthesised with random-effects methods using REML estimation and Knapp–Hartung-type inference [22,23], and between-cohort heterogeneity was summarised with standard meta-analytic metrics [24]. Full estimates are retained in Table S9 and Supplementary Figure S1 and do not replace the three-outcome Model 1/Model 2 analysis. Exploratory HRS polygenic score effect-modification estimates are reported in Table S10.

## 3. Results

### 3.1. Study Population and Observed Follow-Up

**Figure 1:**
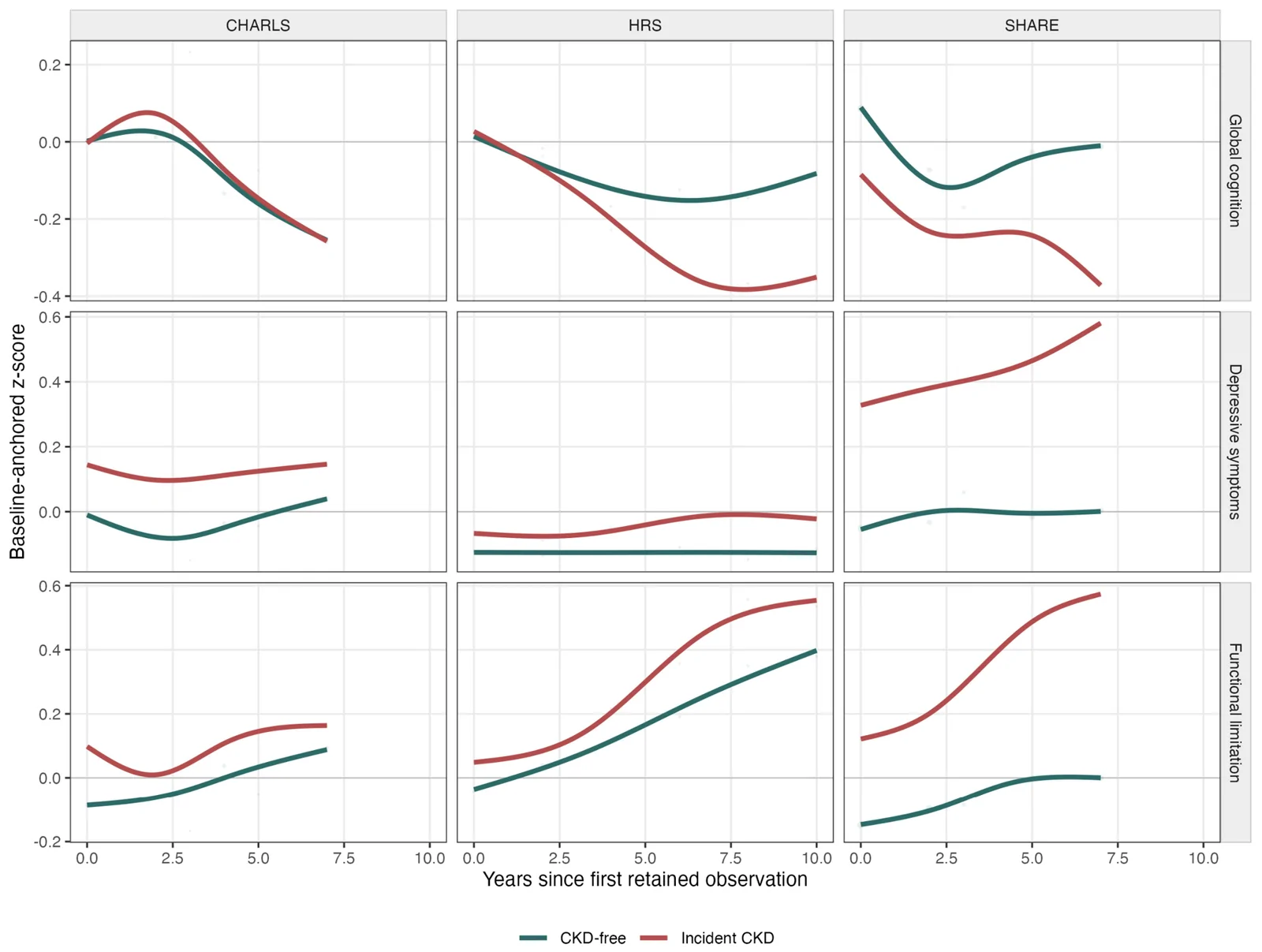
Observed three-domain trajectories by cohort and CKD group. Points are visit-level means for global cognition, depressive symptoms and functional limitation. Curves are weighted descriptive smooths for visualisation only and are not the inferential mixed models.

The analytic cohort comprised 75,044 participants: 72,564 remained free of CKD, and 2480 developed incident CKD during follow-up. Cohort components were CHARLS—11,794 participants, 305 incident CKD; HRS—4664 participants, 1707 incident CKD; and SHARE—58,586 participants, 468 incident CKD. Retained outcome visits contributed 209,403 cognition, 214,968 depressive symptom and 251,894 functional limitation records. The final source-specific counts, under the Chronic Kidney Disease Epidemiology Collaboration (CKD-EPI) equations, were CHARLS—eGFR-creatinine (CKD-EPI 2021): 163/5425; CHARLS—self-report supplement: 142/6369; HRS—eGFR-cystatin-C (CKD-EPI 2012): 1707/4664; and SHARE—self-report: 468/58,586. Thus, HRS contributed 68.8% of incident cases despite representing a much smaller cohort than SHARE, reflecting both ascertainment source and cohort composition rather than a common CKD prevalence estimate. Table 1 summarises baseline characteristics, covariates and unadjusted descriptive group comparison *p* values by CKD status during follow-up. Figure 1 shows observed visit-level mean trajectories for global cognition, depressive symptoms and functional limitation by cohort and CKD group; the curves are descriptive smooths for visualisation only and are not the inferential mixed models. Detailed flow, including the exclusion of 3968 participants with prevalent CKD, is reported in Table S1 and Supplementary Figure S2; kidney source ascertainment and final source-specific counts are reported in Table S2.

### 3.2. Primary Longitudinal Associations Around Incident CKD

**Figure 2:**
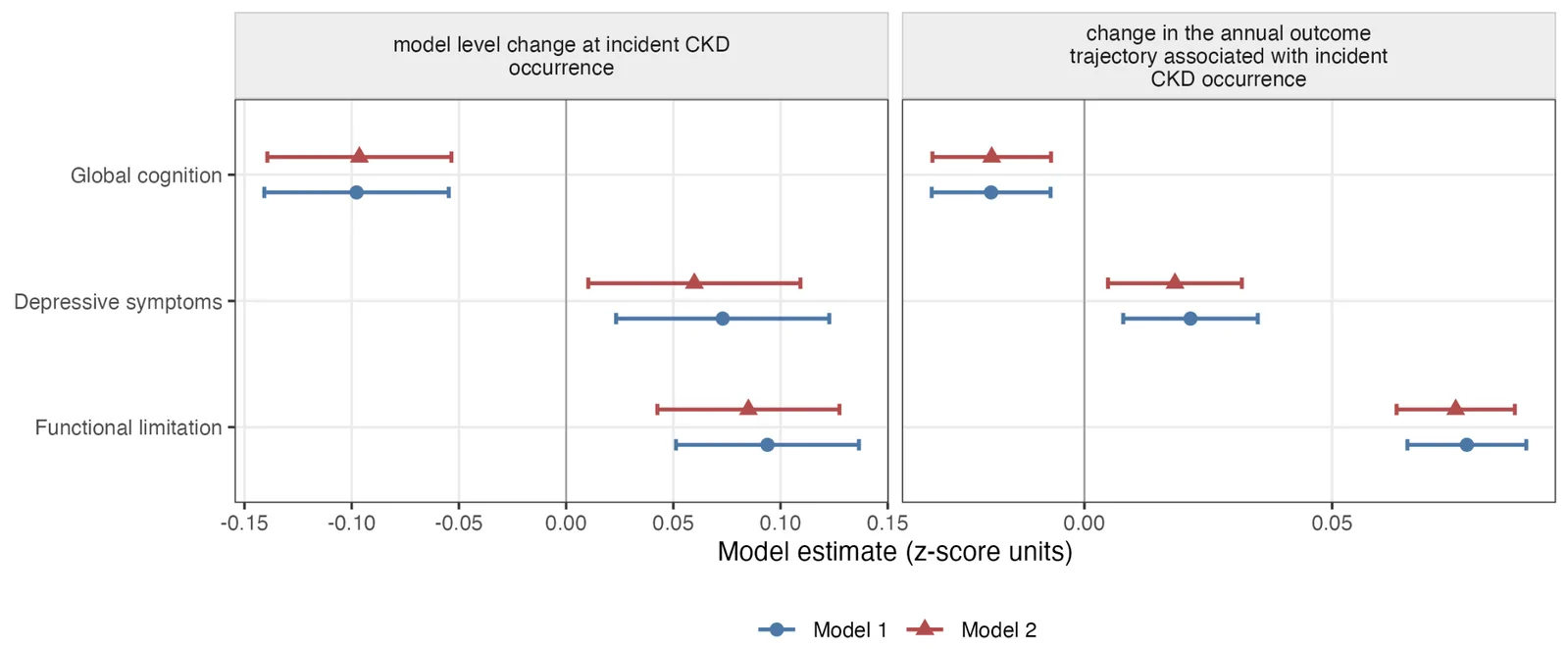
Forest plot of Rubin-pooled CKD model terms for three primary outcomes. Model 1 and Model 2 estimates are shown for the model-level change at incident CKD occurrence and for the change in the annual outcome trajectory associated with incident CKD occurrence. Estimates are in standardised outcome units per year where applicable.

In fully adjusted Model 2, we estimated the change in the annual outcome trajectory associated with incident CKD occurrence for each co-primary domain. The estimates were global cognition, −0.019 (−0.031 to −0.007) (BH q = 0.003); depressive symptoms, 0.018 (0.005 to 0.032) (BH q = 0.008); and functional limitation, 0.075 (0.063 to 0.087) (BH q < 0.001). All are expressed in standardised outcome units per year. These coefficients quantify within-incident change relative to continuation of the pre-CKD trajectory; they are not annual trajectory contrasts versus CKD-free participants. Figure 2 and Table 2 provide the complete Model 1/Model 2 term grid. The separate model-level change coefficient at incident CKD occurrence is retained there and is not interpreted as an instantaneous causal effect. In biomarker-defined CHARLS plus HRS, the corresponding estimates were global cognition −0.019 (95% CI −0.034 to −0.005); depressive symptoms 0.015 (95% CI 0.001 to 0.030); and functional limitation 0.086 (95% CI 0.068 to 0.103). In HRS cystatin-C alone, they were global cognition −0.013 (95% CI −0.028 to 0.002); depressive symptoms 0.016 (95% CI 0.002 to 0.030); and functional limitation 0.086 (95% CI 0.066 to 0.107). The HRS-only cognition interval included zero, whereas depression and functional limitation retained adverse estimates. Sensitivity designs and comparability classes are shown in Table S3; analyses retaining the primary covariance and pooling structure, source-restricted analyses, cohort-specific analyses and clinical translations are in Table S4 and Supplementary Figure S3; marginal weighted analyses remain in Table S5.

### 3.3. Absolute Trajectories Around Incident CKD Occurrence and Cumulative Trajectory Change

**Figure 3:**
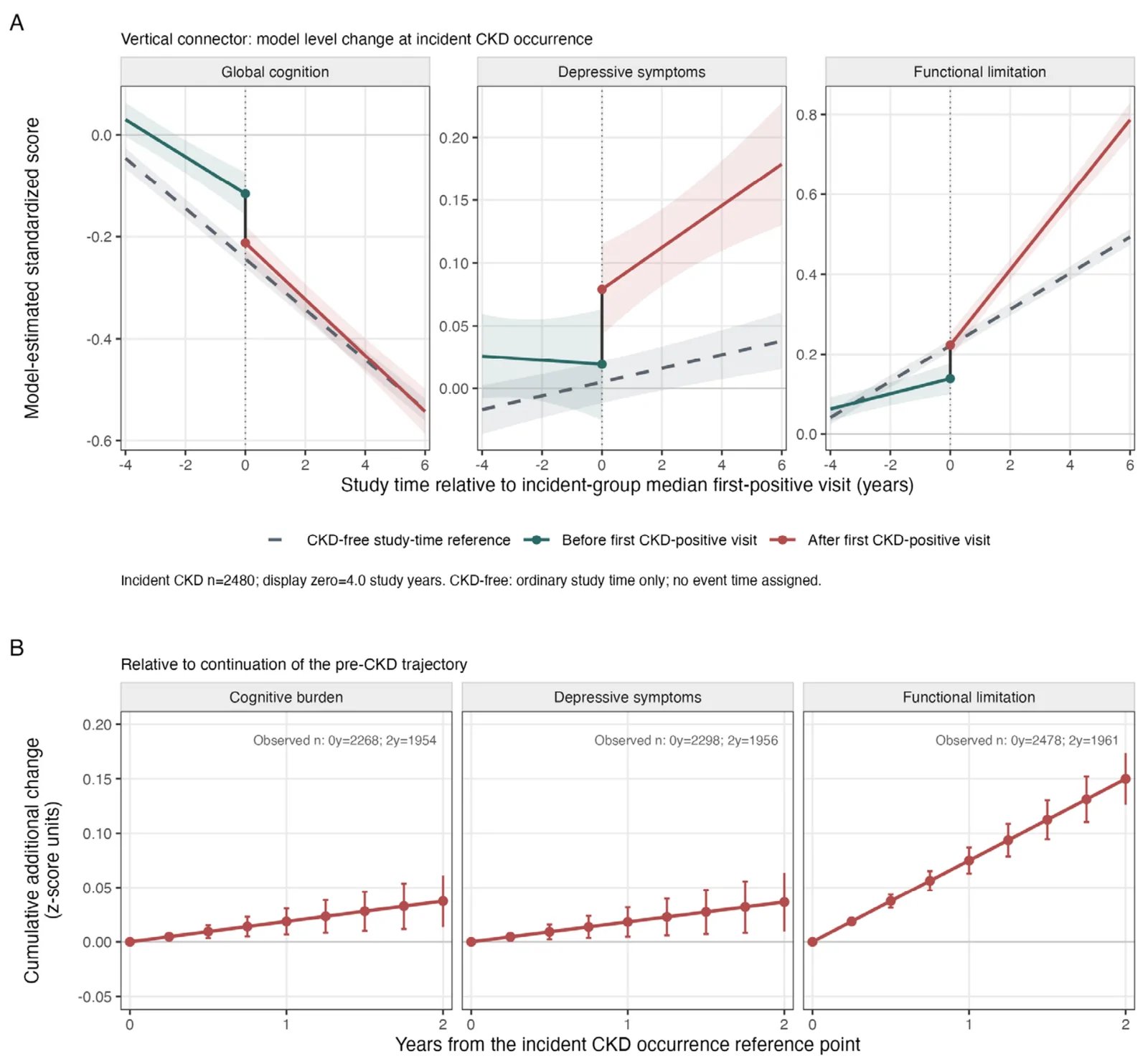
Incident-CKD-centred Model 2 display. Panel (**A**) shows Rubin-pooled adjusted absolute trajectories, standardised to the incident-participant baseline covariate and cohort distribution. Solid teal and red lines with translucent bands denote the before- and after-first CKD-positive visit estimates and pointwise 95% confidence intervals; the dashed gray line and gray band denote the CKD-free ordinary study-time reference and its pointwise 95% confidence interval. The gray dotted vertical line marks the incident group’s median first-positive study time as a shared display origin; CKD-free participants are not assigned an event time. The vertical connector denotes the model-level change at occurrence. Panel (**B**) shows burden-oriented cumulative additional change relative to projected continuation of the incident group’s pre-CKD trajectory (Δ(t) = t × β_trajectory change_); cognition is sign-reversed only here. The horizontal gray line denotes zero additional change, and whiskers are pointwise 95% confidence intervals. Estimates and variances were Rubin-pooled across imputations; intervals are descriptive, not separate hypothesis tests.

Figure 3, Panel A shows incident CKD trajectory segments around CKD occurrence alongside a CKD-free ordinary study time reference on a shared display axis. The origin is derived from the incident group’s median observed study time at incident CKD occurrence for visualisation only and does not assign an event time to CKD-free participants. The vertical connector joins the two incident CKD trajectory segments only and represents the Model 2 level change at incident CKD occurrence. Panel B places the primary trajectory change estimate on a common burden scale and shows burden-oriented cumulative additional change relative to projected continuation of the incident group’s pre-CKD trajectory, with Rubin-pooled pointwise 95% confidence intervals. The displayed quantity is Δ(t) = t × β_trajectory change_, with cognition sign-reversed only for the burden display. At year 2 on the CKD-occurrence-centred display axis, the estimated additional burden in Panel B was cognitive burden, 0.037 (95% CI 0.013 to 0.061); depressive symptoms, 0.037 (95% CI 0.010 to 0.064); and functional limitation, 0.150 (95% CI 0.126 to 0.174). Δ(t) is evaluated at the unrounded pooled coefficient rather than at the rounded value printed above, so the 2-year cognitive burden is 0.037 rather than the 0.038 obtained by doubling −0.019. At 5 years, the unreversed standardised cumulative changes were global cognition −0.094 (95% CI −0.153 to −0.034); depressive symptoms 0.091 (95% CI 0.024 to 0.159); and functional limitation 0.375 (95% CI 0.315 to 0.435). These fixed horizon intervals are linear descriptive transforms of the pooled coefficient; no pointwise hypothesis tests were performed, no cross-cohort raw score was constructed and no validated minimal clinically important difference (MCID) or age-equivalent was available. Cognition’s 0-year value in Panel B is structural. Panel A is a population-average model display, not an individual trajectory or an instantaneous causal effect.

### 3.4. Exploratory Joint Trajectory Classes

**Figure 4:**
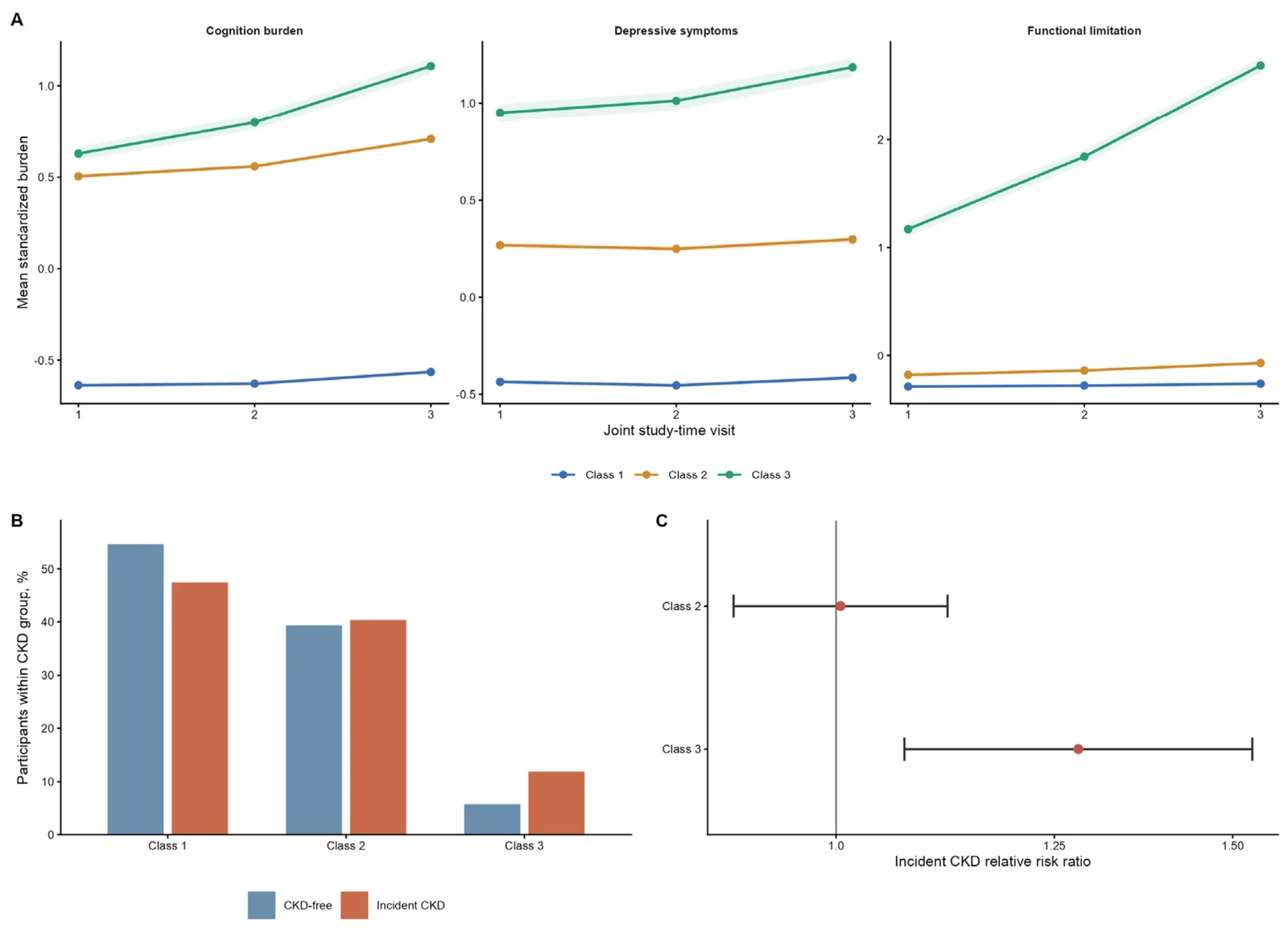
Exploratory joint study-time trajectory classes. Panel (**A**) shows three-domain class profiles; panel (**B**) shows class distributions within incident CKD and CKD-free groups; panel (**C**) shows Rubin-pooled, adjusted relative risk ratios for class membership with the null at 1.0 marked. Classes are aligned to ordinary study time, not to incident CKD occurrence.

The prespecified exploratory study-time module selected three three-domain classes (Figure 4; Table S6). Incident CKD was associated with a higher relative risk of membership in the severe versus lower-burden class (RRR 1.281, 95% CI 1.072 to 1.530; Holm *p* = 0.013), whereas the intermediate-versus-lower comparison was imprecise (RRR 1.005, 95% CI 0.901 to 1.121; Holm *p* = 0.934). These classes summarise early observed study time and are not an occurrence-centred trajectory analysis.

### 3.5. Lagged Visit-Detected Outcomes and Sensitivity Analyses

**Figure 5:**
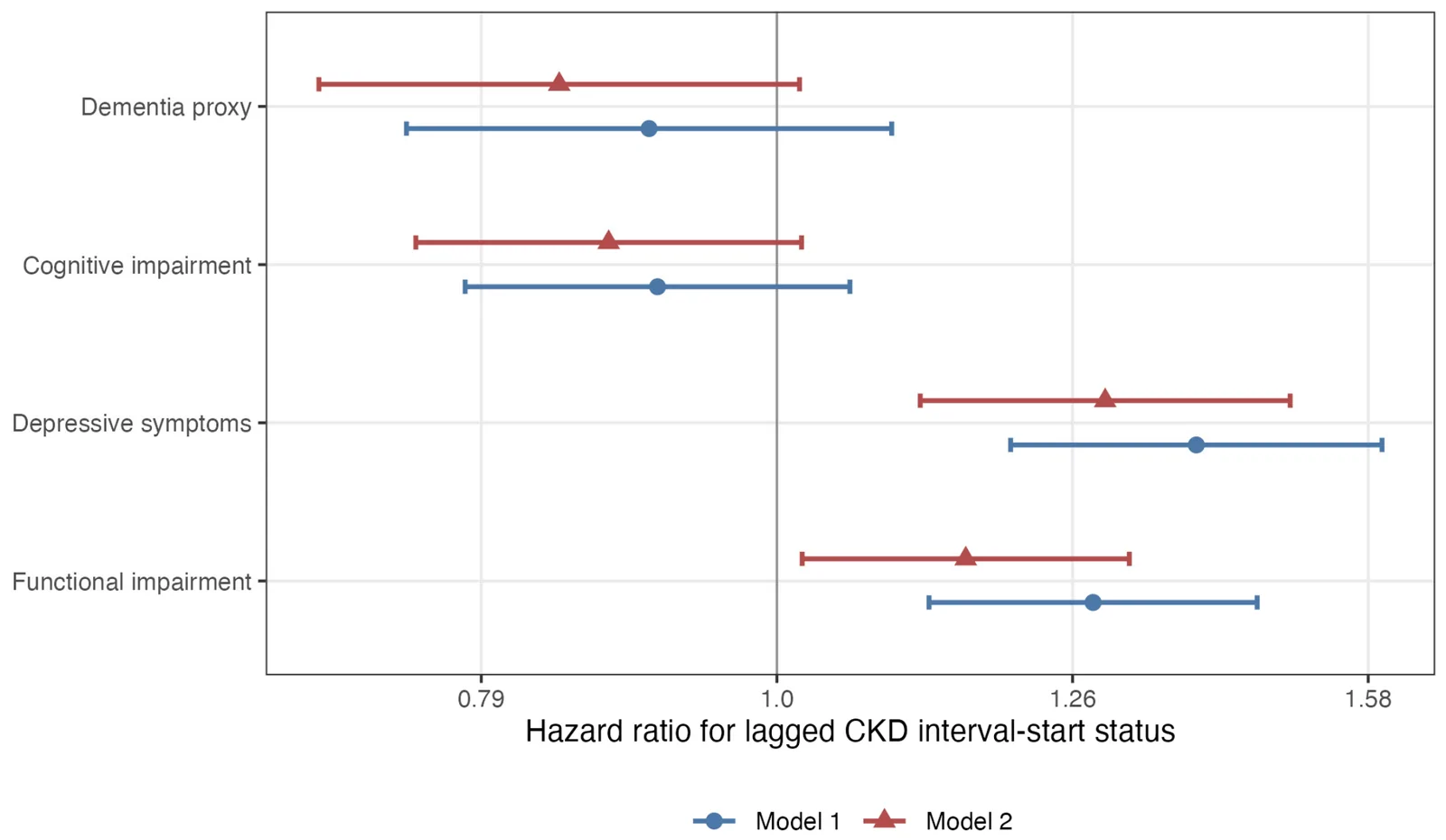
Exploratory Rubin-pooled lagged Cox analyses for four non-frailty, visit-detected proxy endpoints. Model 1 and Model 2 hazard ratios use CKD status at the interval start. Events first detected at a CKD-positive stop visit are not classified as exposed in that interval. The frailty proxy endpoint is reported only in the supplement.

Figure 5 summarises Rubin-pooled lagged Cox estimates for four non-frailty, visit-detected proxy endpoints, with CKD status defined at the interval start and same-wave events left unexposed. These analyses remain exploratory and are not adjudicated diagnoses; full endpoint definitions, estimates and proportional hazards diagnostics are in Table S7. FI10 showed an adverse supplementary annual trajectory contrast of 0.008 (95% CI 0.005 to 0.011; Holm *p* < 0.001) in its outcome-specific sample (Table S8). The supplementary marginal GEE is in Table S9 and Supplementary Figure S1, and HRS polygenic score analyses are in Table S10. None replaces the MICE-plus-Rubin three-outcome primary analysis.

## 4. Discussion

In this three-cohort analysis with cohort-specific multiple imputation and Rubin pooling [14,15,16], incident CKD was associated with directionally and quantitatively distinct changes in three longitudinal outcomes after full adjustment and Benjamini–Hochberg correction. Within the incident CKD group, global cognition declined more rapidly, whereas depressive symptoms and functional limitation showed more adverse annual trajectory changes. The largest absolute standardised point estimate was for functional limitation, consistent with the clinical prominence of physical capacity and sarcopenia risk in kidney disease [6,25,26]. These coefficients describe within-incident trajectory change relative to projected pre-CKD continuation; they should not be read as annual contrasts versus participants who remained free of CKD.

The direction of the findings is consistent with separate research linking CKD to cognitive impairment [4,27,28], depressive symptoms [5,29] and reduced physical functioning [6,25,26] amid a large and ageing CKD burden [1,2,3]. The present contribution is the common event–time analysis of three prespecified co-primary domains, not a composite endpoint. Source-restricted Model 2 results retained adverse depression and functional limitation estimates in biomarker-defined CHARLS plus HRS and in HRS cystatin-C alone; the HRS-only cognition confidence interval included zero. These restrictions support some consistency across ascertainment definitions but do not establish uniform replication across outcomes or cohorts. Differences among standardised point estimates should not be ranked as clinical priorities because the instruments differ in scale, reliability and meaning.

Figure 1 also showed visibly larger descriptive separation in SHARE than in CHARLS or HRS for some domains. Those unadjusted smooths reflect different instruments, visit schedules, case mix and CKD ascertainment and are not cohort-specific tests of the primary trajectory change coefficient. HRS supplied 68.8% of incident cases through repeated cystatin-C ascertainment, whereas SHARE contributed most participants but only 18.9% of incident cases through self-report. These case shares are not model weights and do not establish which cohort determines the pooled estimate.

The cohort-specific Model 2 estimates and leave-one-cohort-out analyses qualify the pooled findings (Table S4a,e; Supplementary Figure S3). Excluding HRS attenuated the annual trajectory change estimates to 0.002 (95% CI −0.032 to 0.035) for depressive symptoms and 0.019 (95% CI −0.008 to 0.045) for functional limitation; both intervals included zero. The corresponding cognition estimate remained adverse at −0.036 (95% CI −0.064 to −0.007). Excluding SHARE retained adverse estimates with nominal evidence across all three domains. In the pooled cohort interaction model, SHARE estimates were −0.029 (95% CI −0.068 to 0.011) for depressive symptoms and −0.016 (95% CI −0.050 to 0.018) for functional limitation. Both pointed in the opposite direction to the pooled adverse associations, with intervals including zero. Thus, magnitude and precision were sensitive to cohort composition, particularly the inclusion of HRS for depression and function. This does not establish that HRS alone generated the associations, but it limits their generalisability across cohorts and health systems.

Several pathways could jointly shape the multidomain pattern, although our design cannot adjudicate among them. Contemporary reviews of the kidney–brain axis emphasise uraemic toxin accumulation, chronic inflammation, endothelial dysfunction, cerebral small-vessel disease, anaemia and tissue hypoxia, blood–brain barrier disruption and metabolic or hormonal disturbance as candidate drivers of cognitive change [27,30,31,32,33,34,35]. Experimental and clinical work links gut-derived and other uraemic solutes to neuronal injury and cognitive phenotypes [31,34], and imaging or permeability studies in advanced kidney disease support blood–brain barrier involvement [36]. Depressive symptoms may share inflammatory, toxin-related, cerebrovascular and illness burden pathways rather than reflecting a purely reactive psychological response [5,29]. Functional limitation is biologically coherent with CKD-associated sarcopenia, protein-energy wasting, inactivity and cardiorenal limitation [6,25,26]. These pathways need not act in lockstep: domain-specific vulnerability, measurement properties and residual confounding can yield quantitatively distinct trajectory changes even when some biology is shared. The present estimates remain adjusted longitudinal associations and should not be read as proof of any single causal mechanism.

Separating the model-level change at incident CKD occurrence from the subsequent trajectory change estimate was essential for interpretation and follows event-centred segmented modelling used in other chronic disease settings [8]. A level contrast could reflect delayed detection, concurrent symptoms, changes in reporting after recognition of illness, or other visit-linked processes as well as underlying disease. Biomarker-defined occurrence is not proof that a participant was informed of a diagnosis, so a diagnosis reaction or “sick role” cannot be asserted. All three level-change coefficients were adverse and statistically significant: global cognition −0.096, depressive symptoms 0.060 and functional limitation 0.085 in standardised units. A concordant adverse level shift across three instruments is compatible with a shared acute biological effect around the time of kidney injury, and equally compatible with visit-linked detection and reporting processes that act on all three instruments at the same assessment. The observed pattern alone cannot separate these explanations. The differing level magnitudes across cognition, mood and function are therefore descriptive model components rather than evidence for one common acute mechanism. Figure 3 makes both components visible, and Panel B isolates cumulative trajectory change from the primary coefficient.

Exploratory study-time trajectory classes provided a complementary group-level view. Incident CKD was associated with a higher relative risk of assignment to the severe rather than lower multisystem burden class (RRR 1.281, 95% CI 1.072 to 1.530; Holm *p* = 0.013). The intermediate-versus-lower contrast did not clearly differ (RRR 1.005, 95% CI 0.901 to 1.121; Holm *p* = 0.934). These classes summarise the first three complete cognition, depression and function visits on ordinary study time; they do not describe an occurrence-centred sequence. Excluding the frailty index from class construction avoided mechanically reinforcing a joint pattern through overlapping deficit accumulation components [7].

Lagged Cox models address a different estimand and should not be expected to reproduce the continuous outcome results. In Model 2, an interval-start CKD-positive status was associated with higher hazards of depressive symptoms (HR 1.291), whereas estimates for cognitive impairment (HR 0.877) and the dementia proxy (HR 0.844) did not meet the nominal threshold. Functional impairment (HR 1.158; nominal *p* = 0.024, Holm *p* = 0.071) showed nominal evidence only and did not remain significant after Holm correction. In particular, a nominal functional-impairment hazard ratio can coexist with a strong continuous functional trajectory change estimate because gradual worsening may be detected by a continuous scale before crossing a dichotomised event threshold.

Strengths include the large combined sample, three international ageing cohorts with established profiles [11,12,13], retention of each cohort’s locked CKD definition based on contemporary eGFR equations or self-report [9,10,11,12,13] and exclusion of prevalent CKD from the primary contrast. Missing covariates were imputed separately within cohort; every analysis was fitted in all 20 completed data sets; and estimates and variances were combined with Rubin’s rules [14,15,16,17]. The design without pseudo-onset assignment, separate disease covariates, lagged Cox exposure and traceable formal source tables further reduced avoidable mismatches among estimand, analysis and reported results.

Limitations remain. CKD ascertainment differed across cohorts and combined creatinine, cystatin-C and self-report sources [9,10,11,12,13], creating potential differential misclassification and unequal case detection. Visit spacing limited the precision of CKD occurrence and outcome timing, and harmonised measures are comparable constructs rather than identical instruments. Model 2 uses baseline comorbidity indicators. This avoids adjusting for later disease changes that may lie on the CKD pathway, but it cannot control time-varying confounding. The requested time-updated sensitivity analysis was not undertaken, so sensitivity to this adjustment choice remains unassessed. The baseline-only completed files do not establish whether the original cohorts could support a suitably harmonised longitudinal analysis. Selection into repeated follow-up, missing-at-random assumptions for MICE [14,15], residual confounding and measurement error may have influenced the estimates. With only three cohorts, heterogeneity summaries are descriptive. FI14 overlaps the primary domains; FI10 reduces overlap but changes the construct and uses an outcome-specific sample. Death time and cause were unavailable in the locked three-cohort data contract; a Fine–Gray subdistribution hazard model was therefore not estimable, and the reported Cox estimates must not be interpreted as competing-risk-adjusted cumulative incidence effects. Finally, the observational design does not support causal or instantaneous interpretations.

Clinically, these findings support evaluating whether follow-up after incident CKD should extend beyond laboratory kidney measures alone. The fixed horizon translations show the scale of model-implied accumulation: at 5 years, standardised changes were global cognition −0.094 (95% CI −0.153 to −0.034); depressive symptoms 0.091 (95% CI 0.024 to 0.159); and functional limitation 0.375 (95% CI 0.315 to 0.435). Cohort-specific raw score equivalents are provided in Table S4g because the instruments and standard deviations differ; a single pooled raw score, age-equivalent or minimal clinically important difference would be unsupported. In HRS, for example, the annual cognitive trajectory change corresponds to −0.084 points on the harmonised raw score, and to −0.418 points over 5 years, against an anchor standard deviation of 4.470. Whether that magnitude matters clinically depends on the instrument and the care setting, and this study establishes no threshold. What the estimates do support is a testable proposition: the three domains change measurably in the years after incident CKD, and a follow-up schedule confined to estimated glomerular filtration rate and albuminuria would not observe those changes. Surveillance that also attends to cognition, depressive symptoms and functional ability may therefore identify emerging needs earlier. These observational estimates do not show that any particular surveillance schedule or intervention improves outcomes.

Turning that observation into practice requires evidence that the present design cannot supply. A study able to guide post-CKD monitoring would need repeated kidney biomarkers rather than wave-spaced measurement so that CKD occurrence is dated more precisely than the visit interval allows. It would need closer outcome assessment, so that the level change at occurrence can be separated from detection and reporting processes. It would need adjudicated rather than visit-detected endpoints, and recorded death time and cause, without which competing risk estimands remain inaccessible. It would need validated clinical thresholds for each instrument, without which a standardised coefficient cannot be converted into a decision rule. It would also need a design able to distinguish the consequences of kidney disease from the consequences of being investigated for it, for example by comparing participants who crossed the same laboratory threshold but were informed at different times. Until such evidence exists, the appropriate reading of these results is descriptive. Incident CKD marks a period during which cognition, mood and function change adversely; clinicians may reasonably ask about those domains, while researchers establish whether acting on the answers changes outcomes.

## 5. Conclusions

Under cohort-specific MICE and Rubin pooling, incident CKD was associated with directionally and quantitatively distinct changes in cognition, depressive symptoms and functional limitation. The estimates are adjusted longitudinal associations. Joint trajectory classes, lagged event models and frailty index analyses remain exploratory or supplementary.

## Figures and Tables

**Table 1:** Baseline characteristics by CKD status during follow-up in the analytic cohort.

Characteristic	Overall	CKD-Free	Incident CKD	Descriptive *p* Value
Cohort				<0.001
CHARLS	11,794 (15.7)	11,489 (15.8)	305 (12.3)	
HRS	4664 (6.2)	2957 (4.1)	1707 (68.8)	
SHARE	58,586 (78.1)	58,118 (80.1)	468 (18.9)	
Age, years	65.4 (9.0)	65.2 (9.0)	69.5 (7.4)	<0.001
Female, n (%)	42,100 (56.1)	40,685 (56.1)	1415 (57.1)	0.329
Education				<0.001
Low	31,935 (42.6)	31,031 (42.8)	904 (36.5)	
Middle	26,751 (35.6)	25,938 (35.7)	813 (32.8)	
High	16,357 (21.8)	15,594 (21.5)	763 (30.8)	
Body mass index, kg/m^2^	26.6 (4.7)	26.6 (4.7)	27.9 (5.3)	<0.001
Current/former smoking, n (%)	14,630 (19.5)	14,285 (19.7)	344 (13.9)	<0.001
Current alcohol use, n (%)	38,900 (51.8)	37,696 (51.9)	1204 (48.5)	<0.001
Hypertension, n (%)	33,592 (44.8)	32,159 (44.3)	1433 (57.8)	<0.001
Diabetes, n (%)	9781 (13.0)	9279 (12.8)	502 (20.2)	<0.001
Cancer, n (%)	5660 (7.5)	5350 (7.4)	310 (12.5)	<0.001
Lung disease, n (%)	6291 (8.4)	6053 (8.3)	237 (9.6)	0.031
Heart disease, n (%)	11,393 (15.2)	10,816 (14.9)	577 (23.3)	<0.001
Stroke, n (%)	3581 (4.8)	3407 (4.7)	174 (7.0)	<0.001
Arthritis, n (%)	26,563 (35.4)	25,104 (34.6)	1459 (58.8)	<0.001
Number of chronic diseases	1.3 (1.2)	1.3 (1.2)	1.9 (1.3)	<0.001

Note: Values are mean (SD) for continuous variables and n (%) for categorical variables unless otherwise indicated. All participants were CKD-free at the first retained visit; prevalent CKD was excluded. Columns: Overall, all participants in the analytic cohort; CKD-free, remaining free of CKD throughout follow-up; Incident CKD, incident CKD detected during follow-up. Baseline is the first retained visit. Summaries were averaged across 20 MICE completions; counts are rounded, and percentages retain one decimal place. Two-sided *p* values compare Incident CKD with CKD-free and were pooled with mice::D1 using Rubin’s rules. They are unadjusted descriptive associations, not multiplicity-corrected primary inferences, and do not assess baseline balance, causality or prognosis. Number of chronic diseases is descriptive only and is not a model covariate.

**Table 2:** Fully adjusted Model 2 associations of incident CKD with three longitudinal outcomes (Rubin-pooled estimates).

Outcome	Model Term	Estimate (95% CI)	*p* Value	BH q	N Participants	N Observations
Global cognition	Pre-CKD annual slope difference (incident vs. CKD-free)	0.013 (0.003 to 0.023)	0.014		75,044	209,403
Global cognition	Model-level change at incident CKD occurrence	−0.096 (−0.139 to −0.053)	<0.001		75,044	209,403
Global cognition	Change in the annual outcome trajectory associated with incident CKD occurrence	−0.019 (−0.031 to −0.007)	0.002	0.003	75,044	209,403
Depressive symptoms	Pre-CKD annual slope difference (incident vs. CKD-free)	−0.007 (−0.018 to 0.004)	0.213		75,044	214,968
Depressive symptoms	Model-level change at incident CKD occurrence	0.060 (0.010 to 0.109)	0.018		75,044	214,968
Depressive symptoms	Change in the annual outcome trajectory associated with incident CKD occurrence	0.018 (0.005 to 0.032)	0.008	0.008	75,044	214,968
Functional limitation	Pre-CKD annual slope difference (incident vs. CKD-free)	−0.026 (−0.036 to −0.016)	<0.001		75,044	251,894
Functional limitation	Model-level change at incident CKD occurrence	0.085 (0.043 to 0.127)	<0.001		75,044	251,894
Functional limitation	Change in the annual outcome trajectory associated with incident CKD occurrence	0.075 (0.063 to 0.087)	<0.001	<0.001	75,044	251,894

Note: Estimates are from random-intercept REML Model 2 models fitted separately in each of 20 completed data sets and combined with Rubin’s rules. Outcomes are in standardised units (cognition higher—better; depressive symptoms and functional limitation higher—worse). Model terms: Pre-CKD annual slope difference, incident versus CKD-free difference in the pre-occurrence annual slope; Model-level change at incident CKD occurrence, level contrast at the first CKD-positive visit; Change in the annual outcome trajectory associated with incident CKD occurrence, the primary within-incident trajectory change estimate. *p* value is the Wald test for the corresponding coefficient. BH q is the Benjamini–Hochberg-adjusted *p* value for the three primary trajectory change estimates only. N participants and N observations are outcome-specific retained sample sizes. Seven chronic diseases enter Model 2 separately. Frailty index analyses are supplementary and are not shown.

## Data Availability

The datasets analysed during the current study are available from the original cohort providers under controlled-access or registration procedures and are not redistributed by the authors. CHARLS data are available from the CHARLS project team at Peking University (https://charls.pku.edu.cn/ or https://charls.charlsdata.com/) after registration and approval of a data-use application. HRS public-use data are available from the University of Michigan Health and Retirement Study (https://hrsdata.isr.umich.edu/) under the HRS data-use agreement. SHARE data are available from SHARE-ERIC (https://share-eric.eu/) after registration and acceptance of the SHARE conditions of use. Researchers seeking access must apply directly to the respective data providers and comply with each provider’s data-use agreement. The authors obtained access to the CHARLS, HRS and SHARE data under the registration procedures and data-use agreements of each provider; registration is complete for all three cohorts, and the respective conditions of use have been accepted. Analytic code, machine-readable tables, figure sources and package manifests used for this manuscript are provided in the formal submission source package accompanying the article; restricted participant-level microdata are not included. The submission source package contains the formal analysis code, machine-readable tables, figure sources and manifests.

## Supplementary Materials

The following supporting information can be downloaded at: https://www.mdpi.com/article/10.3390/healthcare14193215/s1, Figure S1. Participant flow through the formal analysis. Participant counts are shown at each eligibility stage, including exclusion of prevalent CKD before the main comparison. Covariate missingness was handled by cohort-specific multiple imputation and was not an additional exclusion stage. Figure S2. Cohort-specific change in annual outcome trajectories. Points and horizontal lines show Rubin-pooled Model 2 estimates and 95% confidence intervals across 20 imputations. These inferential estimates and the descriptive Figure 1 smooths answer different questions. Figure S3. Direct three-domain stacked GEE analysis. Panels show Rubin-pooled burden differences associated with incident CKD occurrence, domain-specific slope estimates, and cohort-specific synthesis. This analysis is supplementary and does not replace the three-outcome Model 1/Model 2 results. Table S1. (a). Participant and record flow into the fixed three-outcome analytic cohort. (b). Baseline-covariate missingness before multiple imputation. (c). Cohort-specific MICE completion diagnostics. Table S2. (a). Kidney-data sources and operational incident-CKD rules. (b). Final analytic participants by cohort and locked CKD ascertainment source. Table S3. (a). Questions addressed by each sensitivity analysis. (b). Covariance, pooling, and comparability classification. Table S4. (a). Primary-model-matched sensitivity estimates. (b). Isolated alternative covariance-structure analysis. (c). Cohort-specific and leave-one-cohort-out annual trajectory contrasts. (d). Source-restricted primary-structure Model 2 mixed models. (e). Cohort-specific Model 2 trajectory-change coefficients. (f). Fixed-horizon clinical translation of the primary coefficients. (g). Cohort-specific raw-scale equivalents of the primary coefficients. Table S5. (a). CKD-source restrictions and their limits. (b). Weighting targets and estimation models. (c). Rubin-pooled weighted and source-restricted estimates. (d). Prespecified within-cohort weight truncation across completed data sets. (e). Effective sample size and probability-support summary. Table S6. (a). Candidate class solutions. (K = 2–5). (b). Class distribution by CKD group. (c). Label-matched initialization stability. Table S7. (a). Endpoint definitions and detection-time boundaries. (b). Rubin-pooled lagged Cox estimates. (c). Proportional-hazards diagnostics summarized across 20 completed data sets. Table S8. FI14 and reduced-overlap FI10 annual trajectory contrasts. Table S9. (a). Global three-domain D1 tests. (b). Domain-specific marginal annual trajectory contrasts. Table S10. Rubin-pooled HRS polygenic-score interaction estimates.

## Abbreviations

The following abbreviations are used in this manuscript: ADL, activities of daily living; BH, Benjamini–Hochberg; CES-D, Center for Epidemiologic Studies Depression; CHARLS, China Health and Retirement Longitudinal Study; CI, confidence interval; CKD, chronic kidney disease; CKD-EPI, Chronic Kidney Disease Epidemiology Collaboration; eGFR, estimated glomerular filtration rate; FI10, ten-item frailty index; FI14, fourteen-item frailty index; GEE, generalised estimating equations; HR, hazard ratio; HRS, Health and Retirement Study; IADL, instrumental activities of daily living; MCID, minimal clinically important difference; MICE, multiple imputation by chained equations; REML, restricted maximum likelihood; RRR, relative risk ratio; SHARE, Survey of Health, Ageing and Retirement in Europe.
