# Supplementary material for: Changes in Cognition, Depressive Symptoms and Functional Limitation Before and After Incident Chronic Kidney Disease: A Three-Cohort Longitudinal Study
Source: Healthcare (Basel). 2026 Sep 29;14(19):3215. doi: 10.3390/healthcare14193215 (PMC13665304; doi:10.3390/healthcare14193215)
Supplement: Supplementary file 1 [file healthcare-14-03215-s001.zip › healthcare-4490532-supplementary.pdf]

Supplementary material

Companion to: Changes in Cognition, Depressive Symptoms, and Functional Limitation Before and After Incident Chronic Kidney Disease: A Three-Cohort Longitudinal Study

Guide to the supplementary evidence

The main manuscript reports three prespecified co-primary outcome domains under the Model 1/Model 2 analysis; they are not a composite. The domains address distinct cognitive, psychological and functional constructs; no composite score or weighting scheme was prespecified or validated for this analysis. Benjamini-Hochberg adjustment controls the false discovery rate across the three primary Model 2 trajectory-change tests. This supplement documents analytic flow (Table S1 and Supplementary Figure S1), CKD ascertainment and source-specific counts (Table S2), and alternative design and modelling choices (Tables S3–S5 and Supplementary Figure S2). It then provides supporting detail for the exploratory study-time profiles and lagged event models (Tables S6,S7), followed by related but distinct supplementary analyses (Tables S8–S10 and Supplementary Figure S3).

The participant-specific mixed models provide the primary estimates; no table in this document replaces them. FI14 and FI10 overlap the primary domains and covariates.

The direct stacked GEE estimates a marginal rather than participant-specific contrast, and the HRS polygenic-score analyses are exploratory effect-modification analyses.

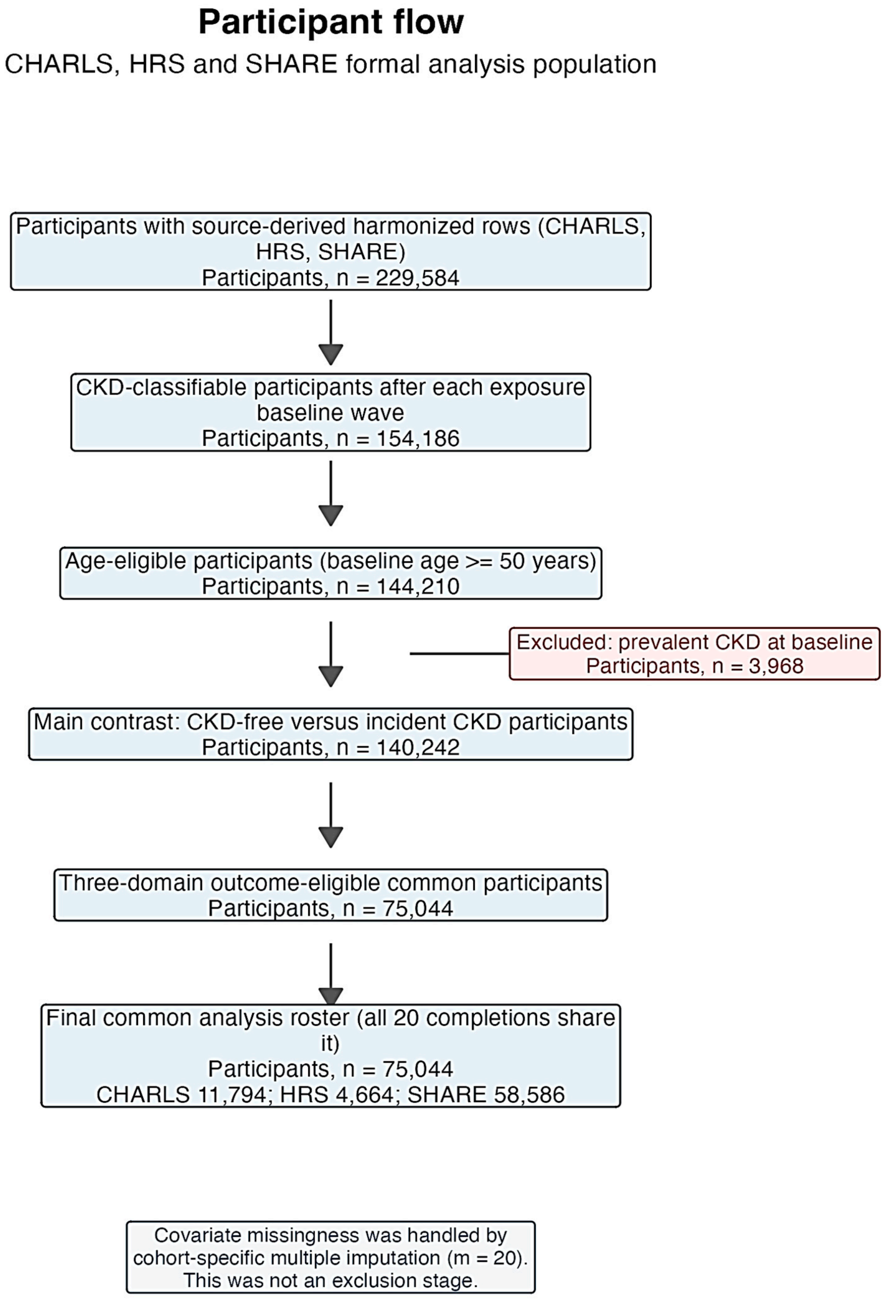

Figure S1. Participant flow through the formal analysis. Participant counts are shown at each eligibility stage, including exclusion of prevalent CKD before the main comparison. Covariate missingness was handled by cohort-specific multiple imputation and was not an additional exclusion stage.

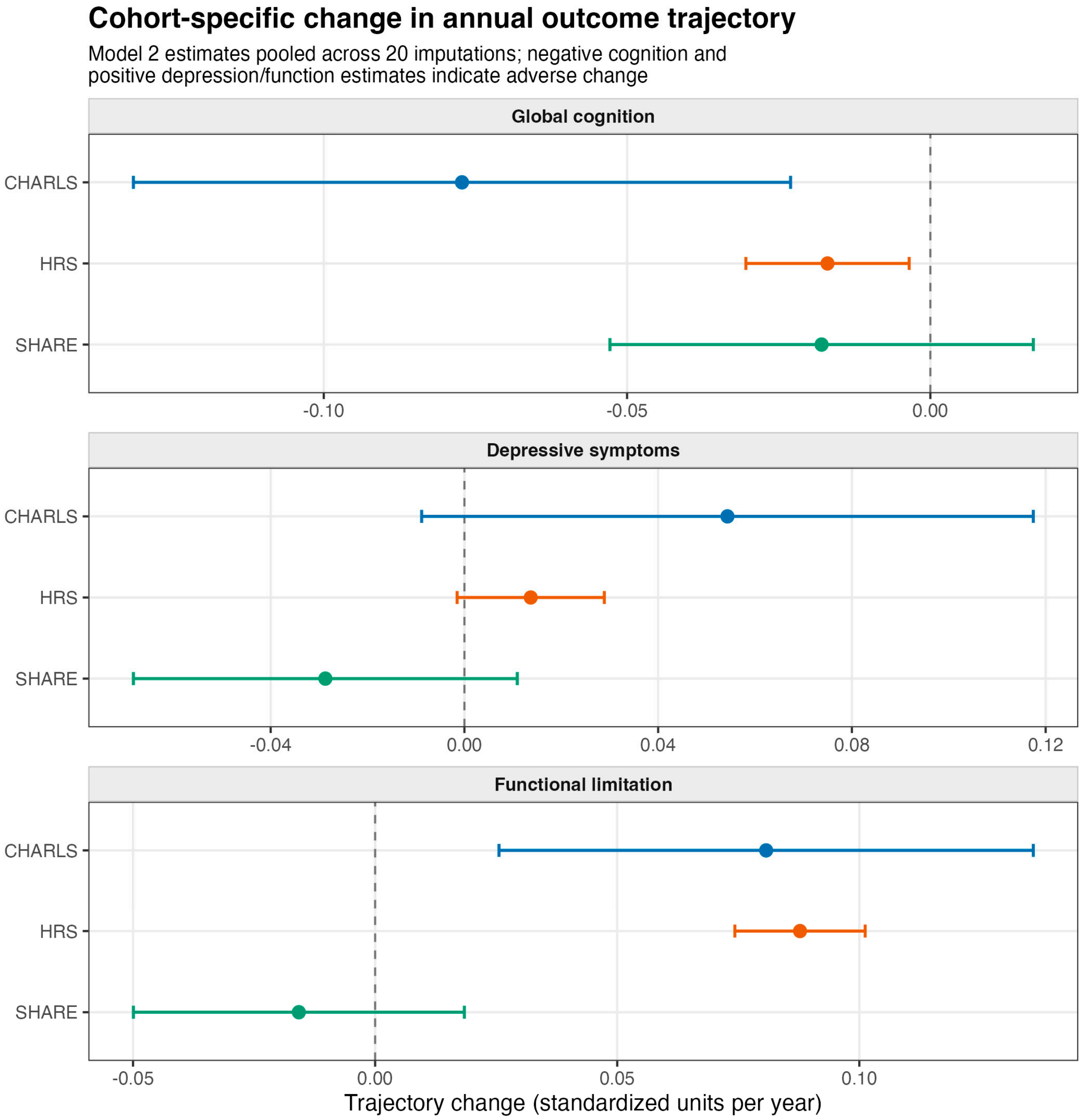

Figure S2. Cohort-specific change in annual outcome trajectories. Points and horizontal lines show Rubin-pooled Model 2 estimates and 95% confidence intervals across 20 imputations. These inferential estimates and the descriptive Figure 1 smooths answer different questions

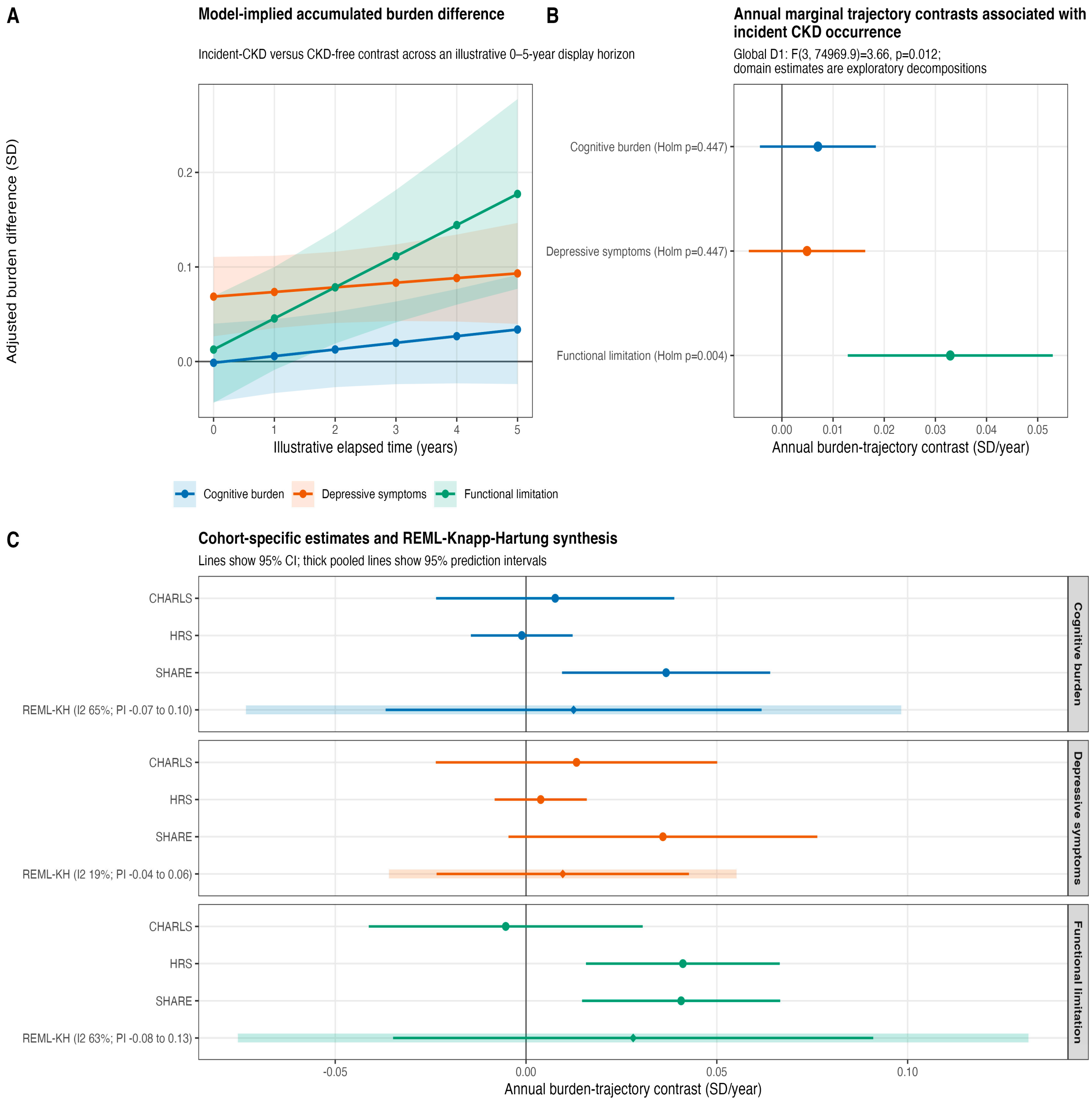

Figure S3. Direct three-domain stacked GEE analysis. Panels show Rubin-pooled burden differences associated with incident CKD occurrence, domain-specific slope estimates, and cohort-specific synthesis. This analysis is supplementary and does not replace the three-outcome Model 1/Model 2 results.

1. Analytic-cohort flow, missingness, and multiple-imputation diagnostics

Purpose. To show which population is represented by the primary estimates and whether missing baseline covariates were handled consistently across cohorts. The fixed primary cohort contains 75,044 participants. The largest baseline-covariate missingness percentages were concentrated in SHARE smoking and alcohol use and CHARLS body mass index; this pattern explains why cohort-specific MICE was necessary.

Table S1 (a). Participant and record flow into the fixed three-outcome analytic cohort

Eligibility stage	Status	Participants	Records	Definition
Participants with source-derived harmonized rows (CHARLS, HRS, SHARE)	Available	229,584	1,643,860	All source-derived rows after layer merge
CKD-classifiable participants after each exposure baseline wave	Available	154,186	527,596	Exclude unclassifiable CKD and waves before each exposure baseline
Age-eligible participants (baseline age >= 50 years)	Available	144,210	496,882	Baseline age >= 50
Prevalent CKD at baseline excluded before the main contrast	Available	3,968	Not supplied	Verified exactly from the current analytic layer: the age-eligible classifiable population is reproduced (144210 participants / 496882 rows) and 144210 - 3968 prevalent-CKD participants = 140242 main-contrast participants; the main-contrast subset is key-identical to the frozen hash-gated preselection roster.
Main contrast: CKD-free versus incident CKD participants	Available	140,242	477,756	CKD-free vs incident CKD
Three-domain outcome-eligible common participants	Available	75,044	283,413	Each domain has at least two observations; incident CKD has a before CKD occurrence and strictly after CKD occurrence observation in every domain
Final common analysis roster (all 20 completions share it)	Available	75,044	252,117	Frozen common roster reproduced by 75044 eligible participants.

Eligibility stage	Status	Participants	Records	Definition
Covariate missingness handled by multiple imputation	Informational	Not supplied	Not supplied	Pre-imputation covariate missingness across the 11 imputation-target variables ranged from 0.00% to 66.27% of participants in the imputation population; missing values were handled by cohort-specific multiple imputation (m = 20). Reported for context; missingness is not an exclusion stage.

Note. Counts are reproduced from the hash-gated participant-flow publication. Exactly 3,968 age-eligible participants with prevalent CKD were excluded before the incident-CKD versus CKD-free comparison. Covariate missingness was handled by cohort-specific MICE and was not an additional participant-exclusion stage. The same flow is shown graphically in Supplementary Figure S1.

Table S1 (b). Baseline-covariate missingness before multiple imputation

Cohort	Variable	Participants	Missing	Missing %
CHARLS	Educational attainment	16,668	4	0.0
CHARLS	Body mass index	16,668	5285	31.7
CHARLS	Smoking status	16,668	492	3.0
CHARLS	Alcohol use	16,668	46	0.3
CHARLS	Hypertension	16,668	103	0.6
CHARLS	Diabetes	16,668	149	0.9
CHARLS	Cancer	16,668	104	0.6
CHARLS	Lung disease	16,668	90	0.5
CHARLS	Heart disease	16,668	106	0.6
CHARLS	Stroke	16,668	72	0.4
CHARLS	Arthritis	16,668	71	0.4
HRS	Educational attainment	9740	1	0.0
HRS	Body mass index	9740	120	1.2
HRS	Smoking status	9740	52	0.5
HRS	Alcohol use	9740	1	0.0
HRS	Hypertension	9740	0	0.0
HRS	Diabetes	9740	0	0.0
HRS	Cancer	9740	0	0.0
HRS	Lung disease	9740	0	0.0
HRS	Heart disease	9740	0	0.0
HRS	Stroke	9740	0	0.0
HRS	Arthritis	9740	0	0.0
SHARE	Educational attainment	113,834	0	0.0
SHARE	Body mass index	113,834	3111	2.7
SHARE	Smoking status	113,834	75,433	66.3
SHARE	Alcohol use	113,834	23,226	20.4
SHARE	Hypertension	113,834	0	0.0
SHARE	Diabetes	113,834	0	0.0

Cohort	Variable	Participants	Missing	Missing %
SHARE	Cancer	113,834	0	0.0
SHARE	Lung disease	113,834	0	0.0
SHARE	Heart disease	113,834	0	0.0
SHARE	Stroke	113,834	0	0.0
SHARE	Arthritis	113,834	0	0.0

Note. Percentages use the cohort-specific age-eligible source population represented in the completion audit. Outcomes, CKD exposure, timing variables, identifiers, and event indicators were not imputed.

Table S1 (c). Cohort-specific MICE completion diagnostics

Cohort	Variables with imputation diagnostics	Final iteration	Chains	Finite diagnostics	Interpretation
CHARLS	Educational attainment, Body mass index, Smoking status, Alcohol use, Hypertension, Diabetes, Cancer, Lung disease, Heart disease, Stroke, Arthritis	20	20	Yes	All reported chains completed with finite diagnostics
HRS	Educational attainment, Body mass index, Smoking status, Alcohol use	20	20	Yes	All reported chains completed with finite diagnostics
SHARE	Body mass index, Smoking status, Alcohol use	20	20	Yes	All reported chains completed with finite diagnostics

Interpretation. Every variable with a registered MICE diagnostic completed 20 iterations across 20 chains with finite diagnostics. Blank source rows for variables without an imputation diagnostic are not reproduced as if they were failed imputations.

2. Cohort-specific CKD ascertainment, chronicity boundary, and source counts

Purpose. To make the heterogeneity of kidney-data sources visible without changing the locked CKD definitions. CHARLS combines creatinine-based classification with self-report supplementation, HRS uses repeated cystatin C-based eGFR, and SHARE uses repeated self-report.

Table S2 (a). Kidney-data sources and operational incident-CKD rules

Cohort	Kidney-data source	Operational incident-CKD rule	Incident participants in source audit	Interpretive boundary
CHARLS	2011 blood biomarker assessment	Creatinine-based definition prioritized; self-report used only when biomarker classification was unavailable	727	Paired biomarker boundary: W1 eGFR <60 is prevalent; W1 eGFR at least 60 and W3 eGFR <60 is incident. Available measurements do not confirm clinical CKD chronicity or persistence for at least 3 months.
CHARLS	2015 blood biomarker assessment	Creatinine-based definition prioritized; self-report used only when biomarker classification was unavailable	727	Paired biomarker boundary: W1 eGFR <60 is prevalent; W1 eGFR at least 60 and W3 eGFR <60 is incident. Available measurements do not confirm clinical CKD chronicity or persistence for at least 3 months.
CHARLS	Harmonized kidney-disease self-report	Creatinine-based definition prioritized; self-report used only when biomarker classification was unavailable	727	Self-report boundary: first observed positive is prevalent and a later first positive is incident; used only when the biomarker classification is unclassifiable. Available reports do not confirm clinical CKD chronicity or persistence for at least 3 months.
HRS	Dried-blood-spot cystatin C biomarker files	Cystatin C-based eGFR definition requiring at least two measurements	3,524	Biomarker boundary: at least two W8-W13 measurements are required; first observed eGFR <60 is prevalent and a later first eGFR <60 is incident. Available measurements do not confirm clinical CKD chronicity or persistence for at least 3 months.

Cohort	Kidney-data source	Operational incident-CKD rule	Incident participants in source audit	Interpretive boundary
SHARE	Harmonized kidney-disease self-report	Repeated kidney-disease self-report across waves 6–9	758	Self-report boundary: first observed positive is prevalent and a later first positive is incident across W6-W9. Available reports do not confirm clinical CKD chronicity or persistence for at least 3 months.

Interpretation. The source audit identifies incident CKD at the applicable biomarker transition or later positive self-report. Available measurements do not uniformly confirm persistence for at least 3 months; the table therefore defines ascertainment, not clinical adjudication. Source-audit incident counts are not the final three-outcome analytic counts because outcome eligibility is applied later.

Table S2 (b). Final analytic participants by cohort and locked CKD ascertainment source

Cohort and ascertainment source	CKD-free	Incident CKD	Total	Incident proportion	Share of all incident cases
CHARLS — eGFR-creatinine (CKD-EPI 2021)	5262	163	5425	3.0%	—
CHARLS — self-report supplement	6227	142	6369	2.2%	—
HRS — eGFR-cystatin C (CKD-EPI 2012)	2957	1707	4664	36.6%	—
SHARE — self-report	58,118	468	58,586	0.8%	—
CHARLS — all ascertainment sources	11,489	305	11,794	2.6%	12.3%
HRS — all ascertainment sources	2957	1707	4664	36.6%	68.8%
SHARE — all ascertainment sources	58,118	468	58,586	0.8%	18.9%
All cohorts — total	72,564	2480	75,044	3.3%	100.0%

Interpretation. These counts use the final fixed three-outcome roster and are stable across all 20 completions. CHARLS contributes both creatinine-defined and self-report-supplemented cases; HRS contributes cystatin-C-defined cases; SHARE contributes self-reported cases. HRS accounts for 68.8% of all incident cases, whereas SHARE accounts for most participants but 18.9% of incident cases. This imbalance is relevant to precision and generalisability.

3. Prespecified sensitivity-analysis design and comparability

Purpose. To distinguish analyses that directly retain the primary Model 2 covariance and pooling contract from analyses that answer a different statistical question. This separation prevents the alternative covariance model or single consensus data set from being presented as equivalent replications of the primary analysis.

Table S3 (a). Questions addressed by each sensitivity analysis

Analysis	Role	Sample / restriction	Model variation	Pooling	Status
Complete three-domain observations at the same visit	Primary-model-matched sensitivity	visits with all three outcomes observed with longitudinal eligibility reapplied	Model 2 fixed-effect structure	Rubin pooling across 20 completed data sets	Completed as specified
Outcome-specific maximum eligible sample	Primary-model-matched sensitivity	maximum eligible sample and observed visits for each outcome	Model 2 fixed-effect structure	Rubin pooling across 20 completed data sets	Completed as specified
Complete baseline covariates before imputation	Primary-model-matched sensitivity	fixed three-outcome analytic cohort participants complete on all baseline covariates before multiple imputation (MI)	Model 2 fixed-effect structure	Rubin pooling across 20 completed data sets	Completed as specified
Excluding the incident-CKD occurrence visit	Primary-model-matched sensitivity	fixed three-outcome analytic cohort excluding the visit at which incident CKD was observed	Model 2 fixed-effect structure	Rubin pooling across 20 completed data sets	Completed as specified
Restricting follow-up to 0–5 study years	Primary-model-matched sensitivity	fixed three-outcome analytic cohort restricted to 0–5 years of study time in both CKD groups	Model 2 without the occurrence-visit level term	Rubin pooling across 20 completed data sets	Completed as specified
Adding a quadratic study-time term	Primary-model-matched sensitivity	fixed three-outcome analytic cohort with quadratic study time and an incident-CKD occurrence trajectory-change basis	Adds a quadratic study-time term	Rubin pooling across 20 completed data sets	Completed as specified
Cohort-by-incident-CKD interaction	Primary-model-matched sensitivity	fixed three-outcome analytic cohort with cohort-by-CKD key-term interactions	Adds cohort-by-incident-CKD key-term interactions	Rubin pooling across 20 completed data sets	Completed as specified

Analysis	Role	Sample / restriction	Model variation	Pooling	Status
Leave-one-cohort-out: excluding CHARLS	Primary-model-matched sensitivity	fixed three-outcome analytic cohort excluding CHARLS	Model 2 fixed-effect structure	Rubin pooling across 20 completed data sets	Completed as specified
Leave-one-cohort-out: excluding HRS	Primary-model-matched sensitivity	fixed three-outcome analytic cohort excluding HRS	Model 2 fixed-effect structure	Rubin pooling across 20 completed data sets	Completed as specified
Leave-one-cohort-out: excluding SHARE	Primary-model-matched sensitivity	fixed three-outcome analytic cohort excluding SHARE	Model 2 fixed-effect structure	Rubin pooling across 20 completed data sets	Completed as specified
Alternative covariance structure	Isolated covariance sensitivity	full fixed three-outcome analytic cohort with the unmodified Model 2 formula; isolated covariance-structure sensitivity	Model 2 fixed-effect structure	Rubin pooling across 20 completed data sets	Completed as specified
Mean/mode single-data-set analysis	Single-data-set sensitivity	fixed three-outcome analytic cohort deterministic mean/mode summary of formal completions	Model 2 fixed-effect structure	Single data set; no Rubin pooling	Completed as specified

Table S3 (b). Covariance, pooling, and comparability classification

Analysis	Comparability class	Random structure	Residual structure	Pooling	Status
Complete three-domain observations at the same visit	Directly comparable with primary Model 2	Participant random intercept	Homoscedastic residuals	Rubin pooling across 20 completed data sets	Completed as specified
Outcome-specific maximum eligible sample	Directly comparable with primary Model 2	Participant random intercept	Homoscedastic residuals	Rubin pooling across 20 completed data sets	Completed as specified
Complete baseline covariates before imputation	Directly comparable with primary Model 2	Participant random intercept	Homoscedastic residuals	Rubin pooling across 20 completed data sets	Completed as specified
Excluding the incident-CKD occurrence visit	Directly comparable with primary Model 2	Participant random intercept	Homoscedastic residuals	Rubin pooling across 20 completed data sets	Completed as specified
Restricting follow-up to 0–5 study years	Directly comparable with primary Model 2	Participant random intercept	Homoscedastic residuals	Rubin pooling across 20 completed data sets	Completed as specified

Analysis	Comparability class	Random structure	Residual structure	Pooling	Status
Adding a quadratic study-time term	Directly comparable with primary Model 2	Participant random intercept	Homoscedastic residuals	Rubin pooling across 20 completed data sets	Completed as specified
Cohort-by-incident-CKD interaction	Directly comparable with primary Model 2	Participant random intercept	Homoscedastic residuals	Rubin pooling across 20 completed data sets	Completed as specified
Leave-one-cohort-out: excluding CHARLS	Directly comparable with primary Model 2	Participant random intercept	Homoscedastic residuals	Rubin pooling across 20 completed data sets	Completed as specified
Leave-one-cohort-out: excluding HRS	Directly comparable with primary Model 2	Participant random intercept	Homoscedastic residuals	Rubin pooling across 20 completed data sets	Completed as specified
Leave-one-cohort-out: excluding SHARE	Directly comparable with primary Model 2	Participant random intercept	Homoscedastic residuals	Rubin pooling across 20 completed data sets	Completed as specified
Alternative covariance structure	Separate covariance sensitivity	Participant random intercept and study-time random slope	Cohort-specific residual scales	Rubin pooling across 20 completed data sets	Completed as specified
Mean/mode single-data-set analysis	Single data set; not Rubin pooled	Participant random intercept	Homoscedastic residuals	Single data set; no Rubin pooling	Completed as specified

Interpretation. Ten sensitivity analyses retained the primary participant random intercept, homoscedastic residuals, REML, and Rubin pooling structure. The alternative covariance analysis and the single-data-set consensus analysis are deliberately classified separately.

4. Robustness of the change in annual outcome trajectory associated with incident CKD occurrence

Purpose. To test whether the direction and precision of the primary estimates of change in the annual outcome trajectory associated with incident CKD occurrence depend on visit completeness, sample definition, occurrence-visit inclusion, follow-up window, time functional form, cohort composition, or covariance assumptions.

Table S4 (a). Primary-model-matched sensitivity estimates

Analysis	Outcome	change in the annual outcome trajectory associated with incident CKD occurrence (95% CI)	P	Covariance
Complete three-domain observations at the same visit	Global cognition	-0.019 (-0.031 to -0.007)	0.002	Participant random intercept; homoscedastic residuals
Complete three-domain observations at the same visit	Depressive symptoms	0.014 (0.000 to 0.028)	0.045	Participant random intercept; homoscedastic residuals
Complete three-domain observations at the same visit	Functional limitation	0.037 (0.025 to 0.049)	<0.001	Participant random intercept; homoscedastic residuals
Outcome-specific maximum eligible sample	Global cognition	-0.019 (-0.031 to -0.007)	0.002	Participant random intercept; homoscedastic residuals
Outcome-specific maximum eligible sample	Depressive symptoms	0.016 (0.004 to 0.028)	0.007	Participant random intercept; homoscedastic residuals
Outcome-specific maximum eligible sample	Functional limitation	0.049 (0.038 to 0.061)	<0.001	Participant random intercept; homoscedastic residuals
Complete baseline covariates before imputation	Global cognition	-0.020 (-0.034 to -0.006)	0.005	Participant random intercept; homoscedastic residuals
Complete baseline covariates before imputation	Depressive symptoms	0.016 (0.001 to 0.031)	0.031	Participant random intercept; homoscedastic residuals
Complete baseline covariates before imputation	Functional limitation	0.088 (0.074 to 0.102)	<0.001	Participant random intercept; homoscedastic residuals
Excluding the incident-CKD occurrence visit	Global cognition	-0.014 (-0.031 to 0.002)	0.083	Participant random intercept; homoscedastic residuals
Excluding the incident-CKD occurrence visit	Depressive symptoms	0.015 (-0.004 to 0.034)	0.131	Participant random intercept; homoscedastic residuals

Analysis	Outcome	change in the annual outcome trajectory associated with incident CKD occurrence (95% CI)	P	Covariance
Excluding the incident-CKD occurrence visit	Functional limitation	0.090 (0.073 to 0.106)	<0.001	Participant random intercept; homoscedastic residuals
Restricting follow-up to 0–5 study years	Global cognition	-0.027 (-0.139 to 0.085)	0.634	Participant random intercept; homoscedastic residuals
Restricting follow-up to 0–5 study years	Depressive symptoms	0.041 (-0.094 to 0.175)	0.552	Participant random intercept; homoscedastic residuals
Restricting follow-up to 0–5 study years	Functional limitation	0.051 (-0.015 to 0.118)	0.129	Participant random intercept; homoscedastic residuals
Adding a quadratic study-time term	Global cognition	-0.020 (-0.044 to 0.003)	0.092	Participant random intercept; homoscedastic residuals
Adding a quadratic study-time term	Depressive symptoms	0.022 (-0.006 to 0.050)	0.123	Participant random intercept; homoscedastic residuals
Adding a quadratic study-time term	Functional limitation	0.053 (0.028 to 0.078)	<0.001	Participant random intercept; homoscedastic residuals
Cohort-by-incident-CKD interaction	Global cognition	-0.077 (-0.131 to -0.023)	0.005	Participant random intercept; homoscedastic residuals
Cohort-by-incident-CKD interaction	Depressive symptoms	0.054 (-0.009 to 0.117)	0.092	Participant random intercept; homoscedastic residuals
Cohort-by-incident-CKD interaction	Functional limitation	0.081 (0.026 to 0.136)	0.004	Participant random intercept; homoscedastic residuals
Leave-one-cohort-out: excluding CHARLS	Global cognition	-0.017 (-0.029 to -0.005)	0.006	Participant random intercept; homoscedastic residuals
Leave-one-cohort-out: excluding CHARLS	Depressive symptoms	0.017 (0.003 to 0.031)	0.017	Participant random intercept; homoscedastic residuals
Leave-one-cohort-out: excluding CHARLS	Functional limitation	0.077 (0.065 to 0.089)	<0.001	Participant random intercept; homoscedastic residuals
Leave-one-cohort-out: excluding HRS	Global cognition	-0.036 (-0.064 to -0.007)	0.015	Participant random intercept; homoscedastic residuals

Analysis	Outcome	change in the annual outcome trajectory associated with incident CKD occurrence (95% CI)	P	Covariance
Leave-one-cohort-out: excluding HRS	Depressive symptoms	0.002 (-0.032 to 0.035)	0.918	Participant random intercept; homoscedastic residuals
Leave-one-cohort-out: excluding HRS	Functional limitation	0.019 (-0.008 to 0.045)	0.172	Participant random intercept; homoscedastic residuals
Leave-one-cohort-out: excluding SHARE	Global cognition	-0.018 (-0.032 to -0.004)	0.014	Participant random intercept; homoscedastic residuals
Leave-one-cohort-out: excluding SHARE	Depressive symptoms	0.017 (0.003 to 0.032)	0.019	Participant random intercept; homoscedastic residuals
Leave-one-cohort-out: excluding SHARE	Functional limitation	0.083 (0.068 to 0.099)	<0.001	Participant random intercept; homoscedastic residuals

Interpretation. All 30 of 30 estimates retained the outcome-specific adverse direction seen in the main analysis; 20 had nominal P<0.05. Precision decreased in the 0–5-year window, and excluding HRS attenuated the depression and functional-limitation estimates. Thus, direction was robust, whereas statistical precision and magnitude were not invariant to every design choice.

Table S4 (b). Isolated alternative covariance-structure analysis

Analysis	Outcome	change in the annual outcome trajectory associated with incident CKD occurrence (95% CI)	P	Interpretation
Alternative covariance structure	Global cognition	-0.020 (-0.034 to -0.006)	0.004	Isolated covariance sensitivity with a study-time random slope and cohort-specific residual scales; not primary-model comparable
Alternative covariance structure	Depressive symptoms	0.023 (0.009 to 0.036)	<0.001	Isolated covariance sensitivity with a study-time random slope and cohort-specific residual scales; not primary-model comparable
Alternative covariance structure	Functional limitation	0.075 (0.057 to 0.092)	<0.001	Isolated covariance sensitivity with a study-time random slope and cohort-specific residual scales; not primary-model comparable

Interpretation. The direction of all three estimates was unchanged, but these values arise from a participant random intercept plus study-time random slope and cohort-specific residual scales. They are a covariance sensitivity, not replacements for the primary Model 2 coefficients.

Table S4 (c). Cohort-specific and leave-one-cohort-out annual trajectory contrasts

Analysis	Outcome	Contrast	Estimate (95% CI)	P
Cohort-by-incident-CKD interaction	Global cognition	Incident-versus-CKD-free annual slope contrast (supplementary): CHARLS	-0.005 (-0.033 to 0.023)	0.743
Cohort-by-incident-CKD interaction	Global cognition	Incident-versus-CKD-free annual slope contrast (supplementary): HRS	-0.003 (-0.013 to 0.006)	0.517
Cohort-by-incident-CKD interaction	Global cognition	Incident-versus-CKD-free annual slope contrast (supplementary): SHARE	-0.028 (-0.054 to -0.002)	0.038
Cohort-by-incident-CKD interaction	Depressive symptoms	Incident-versus-CKD-free annual slope contrast (supplementary): CHARLS	0.015 (-0.019 to 0.049)	0.388
Cohort-by-incident-CKD interaction	Depressive symptoms	Incident-versus-CKD-free annual slope contrast (supplementary): HRS	0.009 (-0.002 to 0.021)	0.117
Cohort-by-incident-CKD interaction	Depressive symptoms	Incident-versus-CKD-free annual slope contrast (supplementary): SHARE	0.030 (-0.002 to 0.061)	0.062
Cohort-by-incident-CKD interaction	Functional limitation	Incident-versus-CKD-free annual slope contrast (supplementary): CHARLS	-0.007 (-0.037 to 0.022)	0.628
Cohort-by-incident-CKD interaction	Functional limitation	Incident-versus-CKD-free annual slope contrast (supplementary): HRS	0.060 (0.050 to 0.070)	<0.001
Cohort-by-incident-CKD interaction	Functional limitation	Incident-versus-CKD-free annual slope contrast (supplementary): SHARE	0.037 (0.017 to 0.057)	<0.001
Leave-one-cohort-out: excluding CHARLS	Global cognition	Incident ckd occurrence trajectory contrast vs ckd free	-0.006 (-0.015 to 0.002)	0.163
Leave-one-cohort-out: excluding CHARLS	Depressive symptoms	Incident ckd occurrence trajectory contrast vs ckd free	0.011 (0.001 to 0.022)	0.039
Leave-one-cohort-out: excluding CHARLS	Functional limitation	Incident ckd occurrence trajectory contrast vs ckd free	0.054 (0.045 to 0.063)	<0.001
Leave-one-cohort-out: excluding HRS	Global cognition	Incident ckd occurrence trajectory contrast vs ckd free	-0.017 (-0.035 to 0.001)	0.068
Leave-one-cohort-out: excluding HRS	Depressive symptoms	Incident ckd occurrence trajectory contrast vs ckd free	0.023 (0.000 to 0.045)	0.047
Leave-one-cohort-out: excluding HRS	Functional limitation	Incident ckd occurrence trajectory contrast vs ckd free	0.022 (0.006 to 0.037)	0.005
Leave-one-cohort-out: excluding SHARE	Global cognition	Incident ckd occurrence trajectory contrast vs ckd free	-0.003 (-0.013 to 0.007)	0.590
Leave-one-cohort-out: excluding SHARE	Depressive symptoms	Incident ckd occurrence trajectory contrast vs ckd free	0.009 (-0.001 to 0.020)	0.081
Leave-one-cohort-out: excluding SHARE	Functional limitation	Incident ckd occurrence trajectory contrast vs ckd free	0.052 (0.040 to 0.064)	<0.001

Interpretation. These contrasts compare annual trajectories associated with incident CKD occurrence with CKD-free trajectories. They are useful for assessing cohort dependence but are not the manuscript-primary change in the annual outcome trajectory associated with incident CKD occurrence.

Table S4 (d). Source-restricted primary-structure Model 2 mixed models

Source restriction	Outcome	change in the annual outcome trajectory associated with incident CKD occurrence (95% CI)	P	Holm P	Participants
Biomarker-defined CHARLS + HRS	Global cognition	-0.019 (-0.034 to -0.005)	0.010	0.020	10,089
Biomarker-defined CHARLS + HRS	Depressive symptoms	0.015 (0.001 to 0.030)	0.039	0.039	10,089
Biomarker-defined CHARLS + HRS	Functional limitation	0.086 (0.068 to 0.103)	<0.001	<0.001	10,089
HRS cystatin-C only	Global cognition	-0.013 (-0.028 to 0.002)	0.084	0.084	4664
HRS cystatin-C only	Depressive symptoms	0.016 (0.002 to 0.030)	0.022	0.045	4664
HRS cystatin-C only	Functional limitation	0.086 (0.066 to 0.107)	<0.001	<0.001	4664

Interpretation. These random-intercept REML models retain the primary trajectory-change estimand and Rubin pooling. The biomarker-defined CHARLS-plus-HRS analysis was directionally adverse for all three domains. In HRS cystatin-C alone, depression and functional limitation remained adverse, whereas the cognition confidence interval included zero. SHARE could not contribute to a biomarker-only analysis because its locked definition is self-report.

Table S4 (e). Cohort-specific Model 2 trajectory-change coefficients

Cohort	Outcome	change in the annual outcome trajectory associated with incident CKD occurrence (95% CI)	P	Holm P	Cohort n / incident n	Model N (pooled interaction model)
CHARLS	Global cognition	-0.077 (-0.131 to -0.023)	0.005	0.012	11,794 / 305	75,044
HRS	Global cognition	-0.017 (-0.030 to -0.003)	0.014	0.027	4664 / 1,707	75,044
SHARE	Global cognition	-0.018 (-0.053 to 0.017)	0.314	0.628	58,586 / 468	75,044
CHARLS	Depressive symptoms	0.054 (-0.009 to 0.117)	0.092	0.092	11,794 / 305	75,044
HRS	Depressive symptoms	0.014 (-0.002 to 0.029)	0.077	0.077	4664 / 1,707	75,044
SHARE	Depressive symptoms	-0.029 (-0.068 to 0.011)	0.156	0.467	58,586 / 468	75,044
CHARLS	Functional limitation	0.081 (0.026 to 0.136)	0.004	0.012	11,794 / 305	75,044
HRS	Functional limitation	0.088 (0.074 to 0.101)	<0.001	<0.001	4664 / 1707	75,044

Cohort	Outcome	change in the annual outcome trajectory associated with incident CKD occurrence (95% CI)	P	Holm P	Cohort n / incident n	Model N (pooled interaction model)
SHARE	Functional limitation	-0.016 (-0.050 to 0.018)	0.366	0.628	58,586 / 468	75,044

Interpretation. These are cohort-specific versions of the manuscript-primary trajectory-change coefficient, not the descriptive Figure 3 smooths or the incident-versus-CKD-free annual slope contrasts in eTable 4C. They are visualised in Supplementary Figure S2. The SHARE depression and functional-limitation point estimates oppose the pooled adverse direction, and both confidence intervals include zero. Together with the attenuation of these two estimates when HRS is excluded (eTable 4B), this limits claims of consistency across cohorts. With only three cohorts, heterogeneity is interpreted descriptively. Cohort n / incident n gives the participants and incident-CKD cases contributed by each cohort; these counts are not model weights. Model N is the number of participants in the single pooled cohort-interaction model from which all nine estimates are drawn; it is shared across rows and is not a per-cohort sample size.

Table S4 (f). Fixed-horizon clinical translation of the primary coefficients

Outcome	Horizon	Cumulative standardised change (95% CI)	Interpretation
Global cognition	2 years	-0.037 (-0.061 to -0.013)	Pointwise linear transform; no separate hypothesis test
Global cognition	5 years	-0.094 (-0.153 to -0.034)	Pointwise linear transform; no separate hypothesis test
Depressive symptoms	2 years	0.037 (0.010 to 0.064)	Pointwise linear transform; no separate hypothesis test
Depressive symptoms	5 years	0.091 (0.024 to 0.159)	Pointwise linear transform; no separate hypothesis test
Functional limitation	2 years	0.150 (0.126 to 0.174)	Pointwise linear transform; no separate hypothesis test
Functional limitation	5 years	0.375 (0.315 to 0.435)	Pointwise linear transform; no separate hypothesis test

Interpretation. Each value equals the pooled annual trajectory-change coefficient multiplied by the stated horizon, with the confidence limits transformed identically. These are pointwise descriptive translations, not additional tests. Cohort-specific raw-score equivalents are reported in eTable 4G, but no universal raw score, age-equivalent or validated minimal clinically important difference was reported.

Time-updated covariate assessment. The seven disease indicators were extracted at baseline, imputed at participant level and replicated at every retained visit. Their constancy across all 20 formal completions therefore reflects the construction of the analysis files. The requested time-updated sensitivity analysis was not undertaken, and no time-updated estimates were generated. The frozen-file audit does not establish that longitudinal disease histories are unavailable in the original cohorts. Such an analysis would require separate harmonisation of those histories, alignment to CKD and outcome visits, and a suitable missing-data strategy. Residual time-varying confounding remains a limitation.

Table S4 (g). Cohort-specific raw-scale equivalents of the primary coefficients

Outcome	Cohort	Quantity	Standardised estimate (95% CI)	Raw-scale equivalent (95% CI)	Anchor SD	Raw-score units
Global cognition	CHARLS	Annual trajectory change	-0.019 (-0.031 to -0.007)	-0.100 (-0.164 to -0.036)	5.337	points on the cohort-specific harmonized raw score per year
Global cognition	CHARLS	Cumulative change, 2 years	-0.037 (-0.061 to -0.013)	-0.200 (-0.328 to -0.072)	5.337	points on the cohort-specific harmonized raw score over the stated horizon
Global cognition	CHARLS	Cumulative change, 5 years	-0.094 (-0.153 to -0.034)	-0.499 (-0.819 to -0.180)	5.337	points on the cohort-specific harmonized raw score over the stated horizon
Global cognition	CHARLS	Level change at occurrence	-0.096 (-0.139 to -0.053)	-0.515 (-0.744 to -0.285)	5.337	points on the cohort-specific harmonized raw score
Global cognition	HRS	Annual trajectory change	-0.019 (-0.031 to -0.007)	-0.084 (-0.137 to -0.030)	4.470	points on the cohort-specific harmonized raw score per year
Global cognition	HRS	Cumulative change, 2 years	-0.037 (-0.061 to -0.013)	-0.167 (-0.274 to -0.060)	4.470	points on the cohort-specific harmonized raw score over the stated horizon
Global cognition	HRS	Cumulative change, 5 years	-0.094 (-0.153 to -0.034)	-0.418 (-0.686 to -0.150)	4.470	points on the cohort-specific harmonized raw score over the stated horizon
Global cognition	HRS	Level change at occurrence	-0.096 (-0.139 to -0.053)	-0.431 (-0.623 to -0.239)	4.470	points on the cohort-specific harmonized raw score
Global cognition	SHARE	Annual trajectory change	-0.019 (-0.031 to -0.007)	-0.215 (-0.352 to -0.077)	11.475	points on the cohort-specific harmonized raw score per year
Global cognition	SHARE	Cumulative change, 2 years	-0.037 (-0.061 to -0.013)	-0.429 (-0.704 to -0.155)	11.475	points on the cohort-specific harmonized raw score over the stated horizon
Global cognition	SHARE	Cumulative change, 5 years	-0.094 (-0.153 to -0.034)	-1.074 (-1.761 to -0.386)	11.475	points on the cohort-specific harmonized raw score over the stated horizon
Global cognition	SHARE	Level change at occurrence	-0.096 (-0.139 to -0.053)	-1.106 (-1.599 to -0.614)	11.475	points on the cohort-specific harmonized raw score

Outcome	Cohort	Quantity	Standardised estimate (95% CI)	Raw-scale equivalent (95% CI)	Anchor SD	Raw-score units
Depressive symptoms	CHARLS	Annual trajectory change	0.018 (0.005 to 0.032)	0.116 (0.030 to 0.201)	6.319	points on the cohort-specific harmonized raw score per year
Depressive symptoms	CHARLS	Cumulative change, 2 years	0.037 (0.010 to 0.064)	0.231 (0.060 to 0.402)	6.319	points on the cohort-specific harmonized raw score over the stated horizon
Depressive symptoms	CHARLS	Cumulative change, 5 years	0.091 (0.024 to 0.159)	0.578 (0.151 to 1.004)	6.319	points on the cohort-specific harmonized raw score over the stated horizon
Depressive symptoms	CHARLS	Level change at occurrence	0.060 (0.010 to 0.109)	0.378 (0.065 to 0.690)	6.319	points on the cohort-specific harmonized raw score
Depressive symptoms	HRS	Annual trajectory change	0.018 (0.005 to 0.032)	0.036 (0.009 to 0.062)	1.957	points on the cohort-specific harmonized raw score per year
Depressive symptoms	HRS	Cumulative change, 2 years	0.037 (0.010 to 0.064)	0.072 (0.019 to 0.124)	1.957	points on the cohort-specific harmonized raw score over the stated horizon
Depressive symptoms	HRS	Cumulative change, 5 years	0.091 (0.024 to 0.159)	0.179 (0.047 to 0.311)	1.957	points on the cohort-specific harmonized raw score over the stated horizon
Depressive symptoms	HRS	Level change at occurrence	0.060 (0.010 to 0.109)	0.117 (0.020 to 0.214)	1.957	points on the cohort-specific harmonized raw score
Depressive symptoms	SHARE	Annual trajectory change	0.018 (0.005 to 0.032)	0.041 (0.011 to 0.072)	2.270	points on the cohort-specific harmonized raw score per year
Depressive symptoms	SHARE	Cumulative change, 2 years	0.037 (0.010 to 0.064)	0.083 (0.022 to 0.144)	2.270	points on the cohort-specific harmonized raw score over the stated horizon
Depressive symptoms	SHARE	Cumulative change, 5 years	0.091 (0.024 to 0.159)	0.207 (0.054 to 0.361)	2.270	points on the cohort-specific harmonized raw score over the stated horizon
Depressive symptoms	SHARE	Level change at occurrence	0.060 (0.010 to 0.109)	0.136 (0.023 to 0.248)	2.270	points on the cohort-specific harmonized raw score

Outcome	Cohort	Quantity	Standardised estimate (95% CI)	Raw-scale equivalent (95% CI)	Anchor SD	Raw-score units
Functional limitation	CHARLS	Annual trajectory change	0.075 (0.063 to 0.087)	0.065 (0.054 to 0.075)	0.862	points on the cohort-specific harmonized raw score per year
Functional limitation	CHARLS	Cumulative change, 2 years	0.150 (0.126 to 0.174)	0.129 (0.109 to 0.150)	0.862	points on the cohort-specific harmonized raw score over the stated horizon
Functional limitation	CHARLS	Cumulative change, 5 years	0.375 (0.315 to 0.435)	0.323 (0.272 to 0.374)	0.862	points on the cohort-specific harmonized raw score over the stated horizon
Functional limitation	CHARLS	Level change at occurrence	0.085 (0.043 to 0.127)	0.073 (0.037 to 0.110)	0.862	points on the cohort-specific harmonized raw score
Functional limitation	HRS	Annual trajectory change	0.075 (0.063 to 0.087)	0.039 (0.032 to 0.045)	0.515	points on the cohort-specific harmonized raw score per year
Functional limitation	HRS	Cumulative change, 2 years	0.150 (0.126 to 0.174)	0.077 (0.065 to 0.089)	0.515	points on the cohort-specific harmonized raw score over the stated horizon
Functional limitation	HRS	Cumulative change, 5 years	0.375 (0.315 to 0.435)	0.193 (0.162 to 0.224)	0.515	points on the cohort-specific harmonized raw score over the stated horizon
Functional limitation	HRS	Level change at occurrence	0.085 (0.043 to 0.127)	0.044 (0.022 to 0.066)	0.515	points on the cohort-specific harmonized raw score
Functional limitation	SHARE	Annual trajectory change	0.075 (0.063 to 0.087)	0.074 (0.062 to 0.086)	0.987	points on the cohort-specific harmonized raw score per year
Functional limitation	SHARE	Cumulative change, 2 years	0.150 (0.126 to 0.174)	0.148 (0.124 to 0.172)	0.987	points on the cohort-specific harmonized raw score over the stated horizon
Functional limitation	SHARE	Cumulative change, 5 years	0.375 (0.315 to 0.435)	0.370 (0.311 to 0.429)	0.987	points on the cohort-specific harmonized raw score over the stated horizon
Functional limitation	SHARE	Level change at occurrence	0.085 (0.043 to 0.127)	0.084 (0.042 to 0.126)	0.987	points on the cohort-specific harmonized raw score

Interpretation. Each raw-scale value is the corresponding standardised quantity multiplied by the cohort-specific anchor standard deviation of the harmonized raw score, computed in the age-eligible CKD-free baseline population of that cohort. These are pointwise descriptive transforms of the pooled coefficient, not separate hypothesis tests. No pooled cross-cohort raw score is reported, because the three instruments differ in content and range. No minimal clinically important difference is reported, because no such threshold is locked for these harmonized scores. No age-equivalent is reported, because that translation would require an age-standardization exercise outside the formal analytic contract.

5. Selection, visit-observation, and CKD-source sensitivity analyses

Purpose. To evaluate two possible sources of bias: selective entry or continued observation in the fixed analytic cohort and differences between biomarker- and self-report-based CKD ascertainment. Because these are marginal GEE estimands, they complement but do not directly validate the conditional primary mixed model.

Table S5 (a). CKD-source restrictions and their limits

Definition source	Included definitions	Interpretive limitation
Biomarker-defined CKD	CHARLS participants classified by the priority creatinine biomarker rule; HRS cystatin-C biomarker definition	SHARE has no biomarker CKD definition in the locked project inputs
Self-report-defined CKD	CHARLS participants requiring self-report supplementation after biomarker priority; SHARE wave 6-9 self-report definition	HRS has no self-report CKD definition in the locked project inputs
All	Locked cohort definitions only; no alternative threshold was created	Source is partly aligned with cohort; only CHARLS contributes to both source strata, so this is a restriction sensitivity rather than a within-cohort source-validation study

Table S5 (b). Weighting targets and estimation models

Weight component	Question addressed	Estimation model
Analytic-cohort selection	Probability of entering the fixed common three-domain participant roster among the complete age-eligible incident-CKD/CKD-free population	cohort-specific baseline logistic regression
Outcome-observation at an eligible visit	Probability that a scheduled harmonized row contributes an observed domain outcome among fixed common-roster participants	cohort- and outcome-specific logistic regression including prior outcome value and prior response
Structural Opportunity	SHARE Wave 7 combined SHARELIFE/regular-panel design: cognition and depression rows without evidence of either regular module are excluded from the visit-response opportunity set; https://www.share-eric.eu/data/data-documentation/waves-overview/wave-7	prespecified survey-module opportunity restriction
Mortality	Death data are absent from the atomic analysis contract; these weights address observed enrollment/roster and visit selection, not mortality	not modeled

Table S5 (c). Rubin-pooled weighted and source-restricted estimates

Analysis	Population or CKD-source restriction	Outcome	Annual marginal trajectory contrast (95% CI)	P	Holm P
Inverse-probability-weighted marginal GEE	Full analytic cohort	Global cognition	-0.019 (-0.034 to -0.005)	0.009	0.026
CKD source-restricted marginal GEE	Biomarker-defined CKD	Global cognition	-0.006 (-0.019 to 0.007)	0.352	0.705
CKD source-restricted marginal GEE	Self-report-defined CKD	Global cognition	-0.017 (-0.040 to 0.006)	0.150	0.150
Inverse-probability-weighted marginal GEE	Full analytic cohort	Depressive symptoms	0.005 (-0.008 to 0.019)	0.429	0.429
CKD source-restricted marginal GEE	Biomarker-defined CKD	Depressive symptoms	0.005 (-0.007 to 0.017)	0.378	0.705
CKD source-restricted marginal GEE	Self-report-defined CKD	Depressive symptoms	0.032 (0.000 to 0.064)	0.048	0.097
Inverse-probability-weighted marginal GEE	Full analytic cohort	Functional limitation	0.034 (0.005 to 0.062)	0.020	0.041
CKD source-restricted marginal GEE	Biomarker-defined CKD	Functional limitation	0.039 (0.014 to 0.063)	0.002	0.006
CKD source-restricted marginal GEE	Self-report-defined CKD	Functional limitation	0.029 (0.007 to 0.052)	0.011	0.033

Interpretation. 4 of 9 marginal contrasts had Holm-adjusted P<0.05. The weighted cognition and functional-limitation contrasts retained adverse directions; source-restricted results were strongest and most consistent for functional limitation. These results show sensitivity to the estimand and source composition rather than a simple pass/fail replication of Model 2.

Table S5 (d). Prespecified within-cohort weight truncation across completed data sets

Outcome	Cohort	Completed data sets	Lower / upper percentile	Weights truncated per data set, median (range)
Depressive symptoms	CHARLS	20	0.01 / 0.99	814 (814–814)
Depressive symptoms	HRS	20	0.01 / 0.99	470 (470–470)
Depressive symptoms	SHARE	20	0.01 / 0.99	3018 (3018–3018)
Functional limitation	CHARLS	20	0.01 / 0.99	846 (845–846)
Functional limitation	HRS	20	0.01 / 0.99	474 (474–474)
Functional limitation	SHARE	20	0.01 / 0.99	3720 (3720–3720)
Global cognition	CHARLS	20	0.01 / 0.99	780 (780–780)

Outcome	Cohort	Completed data sets	Lower / upper percentile	Weights truncated per data set, median (range)
Global cognition	HRS	20	0.01 / 0.99	412 (412–412)
Global cognition	SHARE	20	0.01 / 0.99	3000 (3000–3000)

Table S5 (e). Effective sample size and probability-support summary

Effective sample size (ESS) summarises the precision loss induced by weighting.

Outcome	Cohort	Analysis records	Median ESS (range)	Median ESS / records, %	Probability-support diagnostic
Depressive symptoms	CHARLS	40,669	37,470 (37,431–37,505)	92.1	Selection probabilities below the prespecified support floor were observed; visit-observation probabilities remained within the prespecified support floor
Depressive symptoms	HRS	23,454	17,012 (16,992–17,022)	72.5	Selection probabilities below the prespecified support floor were observed; visit-observation probabilities remained within the prespecified support floor
Depressive symptoms	SHARE	150,845	113,119 (112,737–113,348)	75.0	Selection probabilities below the prespecified support floor were observed; visit-observation probabilities remained within the prespecified support floor
Functional limitation	CHARLS	42,218	38,697 (38,651–38,734)	91.7	Selection probabilities below the prespecified support floor were observed; visit-observation probabilities remained within the prespecified support floor
Functional limitation	HRS	23,685	17,084 (17,077–17,093)	72.1	Selection probabilities below the prespecified support floor were observed; visit-observation probabilities remained within the prespecified support floor
Functional limitation	SHARE	185,991	151,176 (150,710–151,351)	81.3	Selection probabilities below the prespecified support floor were observed; visit-observation probabilities remained within the prespecified support floor

Outcome	Cohort	Analysis records	Median ESS (range)	Median ESS / records, %	Probability-support diagnostic
Global cognition	CHARLS	38,912	35,688 (35,635–35,723)	91.7	Selection probabilities below the prespecified support floor were observed; visit-observation probabilities remained within the prespecified support floor
Global cognition	HRS	20,539	12,932 (12,924–12,941)	63.0	Selection probabilities below the prespecified support floor were observed; visit-observation probabilities remained within the prespecified support floor
Global cognition	SHARE	149,952	120,858 (120,519–121,744)	80.6	Selection probabilities below the prespecified support floor were observed; visit-observation probabilities remained within the prespecified support floor

Interpretation. Weights were truncated at the cohort-specific 1st and 99th percentiles. Low selection-probability support was flagged and is reported rather than hidden; visit-observation probabilities remained within the prespecified support floor. Mortality weighting and competing-risk adjustment were not estimable because death time and cause are absent from the three-cohort atomic input contract; no mortality-adjusted or competing-risk estimate was generated.

6. Supporting evidence for the exploratory study-time profiles in Figure 4

Purpose. To document why the three-class solution was selected, how participants were distributed across classes, and whether the solution was sensitive to k-means initialization. This module describes the first three complete ordinary-study-time visits and is not aligned to incident CKD occurrence.

Table S6 (a). Candidate class solutions (K = 2–5)

Classes (K)	Converged	Eligible	Pseudo-BIC	Calinski-Harabasz index	Smallest class, %	Selected
2	Yes	Yes	-927,089	12,868	24.3	No
3	Yes	Yes	-860,916	11,765	6.3	Yes
4	Yes	No	-811,572	10,951	5.0	No
5	Yes	No	-786,891	9507	4.3	No

Table S6 (b). Class distribution by CKD group

CKD group	Trajectory class	Participants	Within-group %
CKD-free	Class 1: Lower multisystem burden	20,680	54.7
Incident CKD	Class 1: Lower multisystem burden	1164	47.5
CKD-free	Class 2: Intermediate multisystem burden	14,914	39.4
Incident CKD	Class 2: Intermediate multisystem burden	990	40.4
CKD-free	Class 3: Severe multisystem burden	2237	5.9
Incident CKD	Class 3: Severe multisystem burden	294	12.0

Table S6 (c). Label-matched initialization stability

Label-matched agreement across initializations is summarised with the adjusted Rand index (ARI).

Selected K	Successful initializations	Minimum ARI	Median ARI	Mean ARI	Maximum ARI	Interpretation
3	20/20	1.000	1.000	1.000	1.000	Label-matched initialization stability

Interpretation. All 20 of 20 initializations succeeded; the median label-matched ARI was 1.000. Cross-imputation ARI is not applicable because the outcome-protected class solution was constructed once before MICE; completed data sets were used for the class-membership regressions.

7. Supporting definitions, estimates, and diagnostics for the exploratory lagged Cox models in Figure 5

Purpose. To define each visit-detected proxy endpoint, present the complete five-endpoint results including supplementary frailty, and show whether proportional-hazards diagnostics support a simple constant hazard-ratio interpretation.

Table S7 (a). Endpoint definitions and detection-time boundaries

Endpoint	Event rule	Interpretation	Detection-time boundary
Dementia proxy	harmonized global cognition z score <= -1.5 and any observed ADL or IADL limitation	Threshold-based proxy requiring low harmonized cognition and an observed ADL or IADL limitation.	First positive follow-up visit among baseline-negative participants; the stop visit is a detection time, not an exact onset time.
Cognitive impairment	harmonized global cognition z score <= -1.5	Threshold-based endpoint derived from harmonized global cognition.	First positive follow-up visit among baseline-negative participants; the stop visit is a detection time, not an exact onset time.
Depressive symptoms	observed scale cutoff: CHARLS Center for Epidemiologic Studies Depression (CES-D) 10-item >=10; HRS CES-D-8 >=3; SHARE EURO-D >=4	Threshold-based endpoint derived from the cohort depressive-symptom scale.	First positive follow-up visit among baseline-negative participants; the stop visit is a detection time, not an exact onset time.
Functional impairment	any observed ADL or IADL limitation	Threshold-based endpoint derived from observed ADL and IADL limitations.	First positive follow-up visit among baseline-negative participants; the stop visit is a detection time, not an exact onset time.
Frailty	14-item deficit-accumulation index >=0.25	Threshold-based endpoint derived from the formal 14-item frailty index.	First positive follow-up visit among baseline-negative participants; the stop visit is a detection time, not an exact onset time.

Table S7 (b). Rubin-pooled lagged Cox estimates

Endpoint	Model	Hazard ratio (95% CI)	P	Holm P	Events
Dementia proxy	Model 1	0.905 (0.749 to 1.094)	0.302	0.448	4268
Cognitive-impairment proxy	Model 1	0.911 (0.784 to 1.059)	0.224	0.448	6438
Elevated depressive-symptom proxy	Model 1	1.386 (1.200 to 1.602)	<0.001	<0.001	14,259
Functional-impairment proxy	Model 1	1.279 (1.126 to 1.454)	<0.001	<0.001	16,147
Frailty proxy	Model 1	1.600 (1.353 to 1.891)	<0.001	<0.001	8528

Endpoint	Model	Hazard ratio (95% CI)	P	Holm P	Events
Dementia proxy	Model 2	0.844 (0.700 to 1.018)	0.076	0.152	4268
Cognitive-impairment proxy	Model 2	0.877 (0.755 to 1.019)	0.087	0.152	6438
Elevated depressive-symptom proxy	Model 2	1.291 (1.118 to 1.491)	<0.001	0.002	14,259
Functional-impairment proxy	Model 2	1.158 (1.020 to 1.316)	0.024	0.071	16,147
Frailty proxy	Model 2	1.594 (1.348 to 1.884)	<0.001	<0.001	8528

Table S7 (c). Proportional-hazards diagnostics summarized across 20 completed data sets

Endpoint	Model	Completed data sets	Median global P	Minimum–maximum global P	P < 0.05, n / 20
Cognitive-impairment proxy	Model 1	20	0.011	0.011–0.011	20 / 20
Cognitive-impairment proxy	Model 2	20	0.035	0.006–0.081	17 / 20
Dementia proxy	Model 1	20	0.007	0.007–0.007	20 / 20
Dementia proxy	Model 2	20	0.030	0.013–0.053	19 / 20
Elevated depressive-symptom proxy	Model 1	20	0.016	0.016–0.016	20 / 20
Elevated depressive-symptom proxy	Model 2	20	0.003	<0.001–0.010	20 / 20
Frailty proxy	Model 1	20	<0.001	<0.001–<0.001	20 / 20
Frailty proxy	Model 2	20	<0.001	<0.001–<0.001	20 / 20
Functional-impairment proxy	Model 1	20	<0.001	<0.001–<0.001	20 / 20
Functional-impairment proxy	Model 2	20	<0.001	<0.001–<0.001	20 / 20

Interpretation. Global proportional-hazards tests had P<0.05 in 196 of 200 completed endpoint-model fits. The hazard ratios should therefore be read as exploratory summary associations rather than assuming time-constant effects for every endpoint. A cause-specific mortality analysis was not estimable because death time and cause are absent from the three-cohort atomic input contract; no cause-specific or mortality-adjusted cox estimate was generated.

8. Supplementary frailty-index trajectory analyses

Purpose. To assess whether the pattern extends to broader deficit accumulation while avoiding promotion of an overlapping construct into the three-outcome primary family.

Table S8. FI14 and reduced-overlap FI10 annual trajectory contrasts

Index	Annual trajectory contrast (95% CI)	P	Holm P	Participants
FI14	0.012 (0.007 to 0.016)	<0.001	<0.001	98,399
FI10	0.008 (0.005 to 0.011)	<0.001	<0.001	123,778

Interpretation. 2 of 2 indices showed an adverse annual trajectory contrast after multiplicity correction. Concordant evidence, when present, supports a broader deficit-accumulation signal, but component overlap with cognition, depression, function, disease, body mass index, and smoking prevents interpretation as an independent fourth primary domain.

9. Supplementary direct three-domain marginal GEE

Purpose. To ask whether incident CKD is associated with a joint marginal burden-trajectory contrast across all three domains. This estimand differs from the participant-specific primary mixed-model coefficient.

Table S9 (a). Global three-domain D1 tests

Working correlation	Role	D1 statistic	df1	df2	P
Independence	Prespecified supplementary analysis	3.66	3	74,970	0.012
Exchangeable	Working-correlation sensitivity	11.61	3	74,970	<0.001

Table S9 (b). Domain-specific marginal annual trajectory contrasts

Working correlation	Domain	Annual marginal trajectory contrast (95% CI)	P	Holm P
Independence	Cognitive burden	0.007 (-0.004 to 0.018)	0.223	0.447
Independence	Depressive symptoms	0.005 (-0.006 to 0.016)	0.398	0.447
Independence	Functional limitation	0.033 (0.013 to 0.053)	0.001	0.004

Interpretation. The prespecified independence-working-correlation global test was P = 0.012; the exchangeable-working-correlation result was P < 0.001. The reference analysis provided evidence of a joint marginal association at P < 0.05, but in either case it is not a direct validation or replacement of the conditional primary LMM.

10. Exploratory HRS polygenic-score effect modification

Purpose. To explore whether CKD or eGFR polygenic scores modify prespecified level or trajectory contrasts within HRS. These analyses do not alter the three-cohort primary estimand. The table reports one row per polygenic score (PGS), outcome and prespecified interaction.

Table S10. Rubin-pooled HRS polygenic-score interaction estimates

PGS	Outcome	Prespecified interaction	Estimate (95% CI)	P	Holm P	Participants
CKD PGS	Depressive symptoms	PGS interaction with the model level change at incident CKD occurrence	-0.027 (-0.081 to 0.026)	0.318	1.000	4217
CKD PGS	Depressive symptoms	PGS interaction with the change in the annual outcome trajectory associated with incident CKD occurrence	-0.010 (-0.025 to 0.005)	0.173	1.000	4217
CKD PGS	Depressive symptoms	PGS interaction with the pre-occurrence annual trajectory contrast	0.008 (-0.004 to 0.020)	0.194	1.000	4217
CKD PGS	Functional limitation	PGS interaction with the model level change at incident CKD occurrence	0.021 (-0.059 to 0.102)	0.601	1.000	4217
CKD PGS	Functional limitation	PGS interaction with the change in the annual outcome trajectory associated with incident CKD occurrence	-0.000 (-0.022 to 0.022)	0.989	1.000	4217
CKD PGS	Functional limitation	PGS interaction with the pre-occurrence annual trajectory contrast	0.001 (-0.017 to 0.019)	0.895	1.000	4217
CKD PGS	Global cognition	PGS interaction with the model level change at incident CKD occurrence	0.023 (-0.033 to 0.080)	0.417	1.000	4217
CKD PGS	Global cognition	PGS interaction with the change in the annual outcome trajectory associated with incident CKD occurrence	0.006 (-0.010 to 0.022)	0.436	1.000	4217
CKD PGS	Global cognition	PGS interaction with the pre-occurrence annual trajectory contrast	-0.006 (-0.020 to 0.007)	0.358	1.000	4217

PGS	Outcome	Prespecified interaction	Estimate (95% CI)	P	Holm P	Participants
eGFR PGS	Depressive symptoms	PGS interaction with the model level change at incident CKD occurrence	-0.000 (-0.052 to 0.051)	0.985	1.000	4217
eGFR PGS	Depressive symptoms	PGS interaction with the change in the annual outcome trajectory associated with incident CKD occurrence	0.011 (-0.003 to 0.025)	0.117	0.883	4217
eGFR PGS	Depressive symptoms	PGS interaction with the pre-occurrence annual trajectory contrast	-0.006 (-0.017 to 0.005)	0.309	1.000	4217
eGFR PGS	Functional limitation	PGS interaction with the model level change at incident CKD occurrence	0.047 (-0.030 to 0.124)	0.232	1.000	4217
eGFR PGS	Functional limitation	PGS interaction with the change in the annual outcome trajectory associated with incident CKD occurrence	0.020 (-0.001 to 0.041)	0.066	0.592	4217
eGFR PGS	Functional limitation	PGS interaction with the pre-occurrence annual trajectory contrast	-0.014 (-0.031 to 0.003)	0.110	0.883	4217
eGFR PGS	Global cognition	PGS interaction with the model level change at incident CKD occurrence	0.014 (-0.039 to 0.068)	0.603	1.000	4217
eGFR PGS	Global cognition	PGS interaction with the change in the annual outcome trajectory associated with incident CKD occurrence	0.003 (-0.012 to 0.018)	0.704	1.000	4217
eGFR PGS	Global cognition	PGS interaction with the pre-occurrence annual trajectory contrast	-0.003 (-0.016 to 0.009)	0.609	1.000	4217

Interpretation. None of the 18 prespecified interactions remained significant after Holm correction (n=0). The HRS genetic analyses therefore provide no multiplicity-adjusted evidence of effect modification and should not be used to explain the primary three-cohort associations.
